# Heterogeneity in leaf tannin inducibility across woody and non-woody plants: A meta-analysis

**DOI:** 10.1016/j.pld.2026.02.007

**Published:** 2026-02-24

**Authors:** Keke Sun, Jianwei Wang, Yanling Zhang, Youzheng Zhang, Lele Liu, Luis Abdala-Roberts, Yaolin Guo

**Affiliations:** a Zhengzhou Tobacco Research Institute of CNTC, Zhengzhou, China; b State Key Laboratory of Satellite Ocean Environment Dynamics, Key Laboratory of Engineering Oceanography, Second Institute of Oceanography, Ministry of Natural Resources, Hangzhou 310012, China; c Key Laboratory of Ecological Prewarning, Protection and Restoration of Bohai Sea, Ministry of Natural Resources, School of Life Sciences, Shandong University, 72 Binhai Road, Qingdao, China; d Universidad Nacional Autónoma de México, Escuela Nacional de Estudios Superiores-Unidad Mérida, Carretera Mérida-Tetiz Km. 4.5, Ucú, Yucatán 97357, Mexico; e School of Renewable Natural Resources, Louisiana State University Agricultural Center, Baton Rouge, LA 70803, USA

**Keywords:** Herbivory, Chemical defenses, Induced defenses, Woodiness, Chemical ecology, Meta-analysis

## Abstract

Tannins are widespread secondary metabolites that can be induced by herbivory or mechanical damage and contribute to defense against herbivores. Reported tannin inducibility varies across taxa and study designs, making it difficult to separate biological drivers from methodological heterogeneity. Because woodiness covaries with longevity and storage and transport capacity, it may shape inducible chemical defenses, but evidence remains mixed. We synthesized 513 effect sizes from 73 studies, quantified tannin inducibility as the log response ratio (ln*RR*), and assessed phylogenetic signal. Using multilevel meta-analytic models, we tested the effect of woodiness and interactions between woodiness and multiple experimental contexts, and related ln*RR* to damage intensity, damage duration, and sampling lag. We found that tannin inducibility was positive but highly heterogeneous, and its phylogenetic signal was weak and inconsistent. Woody taxa showed consistently positive and often statistically detectable inducibility across tannin groups, sampling designs, and induction methods, whereas estimates for non-woody taxa were more variable and often overlapped zero. Moreover, ln*RR* increased with damage intensity in woody plants, whereas it increased with damage duration only in non-woody plants. In conclusion, our synthesis indicates woodiness as an informative axis for leaf tannin inducibility, suggesting that variation in inducible quantitative defenses is not fully explained by phylogenetic relatedness and may reflect broad life history strategy. To move from pattern to mechanism, future studies should pair inducibility estimates with measurements of storage, transport, and allocation traits under standardized damage and sampling protocols, thereby improving comparability across systems and strengthening mechanistic inference.

## Highlights

• We synthesized 513 effect sizes from 73 studies to quantify leaf tannin inducibility (ln*RR*).
• Tannin inducibility was positive overall but highly heterogeneous, with weak phylogenetic signal.
• Woody taxa showed more consistently detectable positive inducibility across tannin groups and study designs.
• These results underscore context dependence in tannin induction and the predictive value of woodiness.

## 1 Introduction

Plants interact with a diverse array of herbivores, ranging from insects to large mammals that threaten plant survival and reproduction and can drive coevolutionary arms races between plants and herbivores (Ehrlich and Raven, 1964; Futuyma and Agrawal, 2009; Endara et al., 2017, Endara et al., 2023). In response, plants have evolved diverse defensive strategies, with chemical defenses as a major component (Howe and Jander, 2008; Mithöfer and Boland, 2012). Plants produce diverse secondary metabolites that reduce tissue palatability and the digestive efficiency of herbivores; these compounds can be expressed constitutively or induced following damage (Karban and Baldwin, 2007; Mithöfer and Boland, 2012; Agrawal and Weber, 2015). Unlike constitutive chemical defenses, which are typically expressed in the absence of herbivory, induced defenses are triggered by tissue damage caused by herbivore feeding or mechanical wounding, allowing plants to deploy potentially costly metabolites when they are most needed (Karban, 2011; Huot et al., 2014; Kant et al., 2015; Erb and Reymond, 2019). However, induced responses vary widely across taxa in magnitude, duration, and specificity, and their expression depends on both biotic and abiotic factors (Rasmann et al., 2014; Agrawal and Weber, 2015; Erb and Reymond, 2019).

Among the diverse secondary metabolites involved in plant–herbivore interactions, tannins are particularly important due to their widespread occurrence and well-documented anti-herbivore effects (Feeny, 1970, 1976; Bernays et al., 1989; Endara and Coley, 2011). As abundant, carbon-based polyphenols that often function as quantitative defenses, tannins are expected to be sensitive to resource availability and plant storage capacity, both of which can covary with growth form and life history (Feeny, 1976; Herms and Mattson, 1992; Barbehenn and Constabel, 2011; Salminen and Karonen, 2011). Hydrolysable and condensed tannins can bind proteins in the herbivore digestive tract, reducing the digestibility and nutritional value of plant tissues (Barbehenn and Martin, 1994; Barbehenn and Constabel, 2011). They may also act through oxidation in the alkaline environment of insect midguts, with oxidation products contributing to gut tissue damage (Appel, 1993; Salminen and Karonen, 2011). By altering herbivore gut physiology and deterring feeding, increased tannin concentrations may reduce herbivore pressure (Appel, 1993; Barbehenn and Constabel, 2011). Notably, many plants exhibit inducible tannin defenses, characterized by a rapid increase in tissue tannin levels following herbivore attack or mechanical wounding (Stevens and Lindroth, 2005; Constabel et al., 2014). Variation in tannin inducibility suggests that life history and ecological context may determine whether tannin induction is detectable and consistent across systems (Herms and Mattson, 1992; Häring et al., 2007; Endara et al., 2017, 2023).

Woodiness is a key growth form axis associated with life history traits that can structure resource allocation to defenses and plant–herbivore interactions (Coley et al., 1985; Herms and Mattson, 1992; Haukioja and Koricheva, 2000; Defossez et al., 2018). Because woodiness covaries with plant size, longevity, and reserve capacity, it is expected to influence inducible investment and potential trade-offs with constitutive allocation through differences in carbon storage, tissue turnover, and vascular integration (Coley et al., 1985; Herms and Mattson, 1992; Endara and Coley, 2011; Moreira et al., 2014). In many systems, herbivory regimes may differ by growth form, such that non-woody plants are often less apparent and experience episodic but variable damage, whereas woody plants may face more chronic or repeated attack. These patterns are often expected to favor greater inducible defenses in non-woody taxa and greater constitutive investment in woody taxa (Feeny, 1976; Coley and Barone, 1996). However, empirical evidence for woodiness-related patterns in tannin induction remains mixed across studies (Eyles et al., 2010; Smilanich et al., 2016; Defossez et al., 2018).

Interpreting variation in induced tannin responses, especially differences associated with growth form, is hampered by heterogeneity in methods and sampling designs across studies, which complicates comparisons and obscures general patterns (Karban and Baldwin, 2007; Ojha et al., 2022). First, assay-dependent estimates of total tannins can obscure shifts because hydrolysable and condensed tannins differ in biosynthesis and chemical properties, translating into divergent induction magnitudes, kinetics, and herbivore guild-specific effects (Barbehenn and Constabel, 2011; Marsh et al., 2020). Second, sampling design and induction method strongly shape inferred magnitudes of induction. Distinguishing damaged from undamaged leaves on damaged plants separates local from systemic signaling, whereas distinguishing preexisting from regrowth leaves helps control for ontogeny and post-damage accumulation (Peters and Constabel, 2002; Karban and Baldwin, 2007; Barton and Koricheva, 2010; Rubert-Nason et al., 2015). Damage intensity and duration, together with sampling lag, shape response trajectories and interact with tissue turnover and storage capacity, which can differ by woodiness (Karban and Baldwin, 2007; Underwood, 2012). Different induction methods, including natural herbivory, mechanical wounding, and chemical elicitors, activate overlapping yet distinct pathways (Karban and Baldwin, 2007; Erb and Reymond, 2019). Because these contrasts are rarely assessed jointly, an integrative framework that explicitly models design choices, context, and their interactions with woodiness is needed to separate biological signal from methodological noise and advance inference on tannin induction (Kant et al., 2015; Mishra et al., 2015; Ojha et al., 2022).

Despite decades of empirical work, it remains unclear whether tannin inducibility differs consistently between woody and non-woody taxa across experimental contexts. Quantitative syntheses focused specifically on tannin induction are rare, and existing evidence is difficult to compare because studies vary in tannin group definitions, sampling designs, and induction methods. Here, we synthesized the literature using a systematic meta-analysis, extracting 513 effect sizes (ln*RR*) from 73 articles to quantify tannin inducibility, capturing proportional chemical change rather than realized resistance or fitness outcomes, and to test whether woodiness and methodological choices jointly shape induction patterns. We placed woodiness (woody vs. non-woody) at the core of our framework and asked three questions: (1) How do induced tannin responses differ between woody and non-woody plants? (2) Does the woody versus non-woody contrast vary among tannin groups? (3) Is the woody versus non-woody contrast contingent on sampling design and induction method?

## 2 Methods

### 2.1 *Literature search*

We conducted a comprehensive literature search to identify empirical studies reporting quantitative changes in leaf tannin concentrations following natural herbivory, experimentally simulated damage, or chemical elicitation. We systematically searched Web of Science, Scopus, and Google Scholar using the following query: ("tannin∗" OR "leaf tannin∗" OR "foliar tannin∗" OR "condensed tannin∗" OR "hydroly∗ tannin∗") AND (induc∗ OR herbivor∗ OR damag∗ OR wound∗ OR defoliat∗ OR jasmon∗ OR "methyl jasmonate∗" OR MeJA OR "salicylic acid") AND (plant∗ OR leaf∗). The final database search was completed on 1 March 2025. To ensure comprehensive coverage, we screened reference lists of relevant publications and conducted forward citation searches for key articles.

All records were screened by titles and abstracts to remove studies irrelevant to tannins or to herbivory-induced responses. Full-text articles were then assessed for eligibility against the following criteria: (1) taxon and tissue: the focal organisms were vascular plants and tannins were quantified in leaf tissue; species-level identification was required, and studies limited to genus or higher ranks or reporting mixed-species composites without species-specific estimates were excluded; measurements on roots, stems, bark, seeds or whole-plant composites were excluded unless a leaf-specific estimate was available; (2) exposure: the study involved natural herbivory (e.g., herbivore feeding) or an explicit simulation of herbivory, including mechanical wounding such as clipping, punching or defoliation, or chemical elicitors linked to defense signaling such as jasmonic acid, methyl jasmonate or salicylic acid; studies focused solely on pathogens or abiotic stress were excluded; (3) outcome and assay: leaf tannin concentration was quantified using tannin-specific methods, accepting condensed tannins, hydrolysable tannins or clearly defined total tannins, while studies reporting only total phenolics without a tannin-specific correction were excluded, and treatment and control had to be analyzed with the same assay and mass basis or be convertible to a common basis; (4) design and controls: a concurrent control or baseline was required to permit quantitative comparison of induced vs. control (baseline) levels, and both between-subject and within-subject designs were eligible when controls were clearly defined; (5) minimum data for effect sizes: sample size, group mean and a measure of dispersion (standard deviation, standard error) had to be available, with values digitized from figures; (6) contextual information: sufficient detail had to be provided to code the induction method and woodiness (woody or non-woody); (7) multiple comparisons: when papers reported several eligible contrasts across treatments, leaf conditions, cohorts, herbivore diet breadth, intensities or time points, each was extracted as a separate effect size; (8) study type: only peer-reviewed English-language research articles were included.

### 2.2 *Data collection*

Database searches identified 3886 records; after title and abstract screening and full-text eligibility assessment, 73 studies met the inclusion criteria and were retained for meta-analysis (Table S1). From each eligible publication, we extracted the following information: (1) study-level metadata; (2) moderator variables (effect-size descriptors); (3) effect-size calculation inputs.

#### 2.2.1 Study-level metadata

For each included publication, we first extracted study-level information to document data sources and to support modeling of non-independence within publications and among species in subsequent analyses. This information included publication year, journal name, and journal impact factor. We also assigned a unique Publication ID to each paper and an Effect ID to each extractable control-to-treatment comparison within a paper to track the within-study structure of multiple effect sizes and to support multilevel random-effects modeling.

#### 2.2.2 Moderator variables

For each effect size, we extracted and coded the associated biological and methodological context variables (moderators) to explain heterogeneity and to conduct subgroup and interaction analyses. Biological moderators included plant taxonomy (family and species), woodiness (woody vs. non-woody), life span (annual vs. perennial), and leaf tannin group (total tannins, hydrolysable tannins, or condensed tannins). Methodological moderators included leaf damage status (damaged leaves, undamaged leaves on damaged plants, or mixed leaves), leaf cohort (preexisting leaves, regrowth leaves, or mixed leaves), and induction method (mechanical induction, herbivore induction, chemical induction, or joint induction).

Plant scientific names were standardized, and synonyms were reconciled to accepted names using Plants of the World Online (POWO; Royal Botanic Gardens, Kew) prior to analysis. Woodiness was coded primarily from growth-form descriptions in the original articles. For borderline growth forms (e.g., subshrubs, partially woody herbs, or lianas) or when information was insufficient, we assigned woodiness only when supported by clear evidence from the original study or an authoritative trait source; otherwise, woodiness was coded as NA. Although non-woody taxa are often herbaceous, we retain “non-woody” as the operational term because “herbaceous” is not always strictly equivalent across primary studies and growth forms. Life span was assigned using species-level information from authoritative floras or trait databases and coded as NA when it could not be determined with confidence. Records using ambiguous compound terms that could not be reliably mapped to a tannin category (e.g., reporting only “phenolics” without tannin-specific information) were excluded to avoid misclassification.

When sufficient information was available, we further coded induction method subtypes. These included mechanical induction subtype (defoliation, pruning, punching), herbivore induction subtype (i.e., herbivore diet breadth; generalist vs. specialist), chemical induction subtype (i.e., chemical elicitor; jasmonic acid [JA] or salicylic acid [SA]), and the composition of joint induction treatments. Mechanical induction subtype was coded based on how tissue was removed or wounded in the primary study: defoliation refers to partial or complete removal of leaf area (e.g., clipping, cutting, or mowing), pruning refers to removal of shoots/branches or larger plant parts beyond leaves, and punching refers to localized wounding without large-scale tissue removal (e.g., hole-punching or piercing). Herbivore induction subtype (i.e., herbivore diet breadth; generalist vs. specialist) was coded only when herbivore identity and/or host-range information was explicitly provided. We classified herbivores as specialists when they were described as mono-/oligophagous or restricted to a narrow host range (e.g., a single genus or family), and as generalists when described as polyphagous or feeding across multiple plant families. Diet-breadth classifications were taken primarily from the primary studies; when descriptions were insufficient to assign diet breadth with confidence, the record was coded as NA. We defined joint induction as the simultaneous application of two or more induction methods within the same control-to-treatment comparison, thereby representing composite induction signals. Such comparisons were first coded as joint induction and, when possible, further classified by joint type (e.g., defoliation + herbivore, defoliation + salivation, defoliation + JA) to distinguish them from single-method induction (mechanical, herbivore, or chemical) and to enable supplementary comparisons. When the components of joint treatments were not clearly described or could not be confirmed as composite induction within a single comparison, the record was retained as joint induction (without further subclassification) and missing information was documented in the dataset.

Additionally, when sufficient information was reported, we extracted three continuous induction-regime variables: damage intensity (the reported percentage of leaf area removed, or the closest comparable metric), damage duration (the length of continuous or repeated damage exposure), and sampling lag (the time elapsed between the end of damage exposure, i.e., the final damage event in repeated treatments, and chemical sampling). All time units were converted to days. Records lacking sufficient information were coded as NA and were not used in regressions involving these variables (see Section 2.4 Statistical analyses).

#### 2.2.3 Effect size calculation inputs

We extracted the raw quantitative information required to calculate ln*RR* and its sampling variance. We extracted group means, sample sizes (n), and measures of uncertainty, preferably standard deviation (SD) and otherwise standard error (SE). When only SE was reported, we converted it to SD using $SD=SE\times \sqrt{n}$. When data were presented only in figures, we digitized means and error bars using PlotDigitizer after calibrating the axes. When a study reported multiple extractable control-to-treatment comparisons (e.g., different damage intensities, durations, sampling lags, or multiple treatment groups), we extracted all eligible comparisons and accounted for dependence among effect sizes within studies using multilevel random-effects models. If the information required for variance-weighted analyses could not be obtained after reasonable conversion (e.g., missing n or uncertainty metrics that could not be inferred from figures or text), that comparison was excluded from subsequent analyses.

### 2.3 *Effect size calculation*

We converted reported tannin data into effect sizes using the log response ratio (ln*RR*) as described by Borenstein (2009), calculated by the following formula:

$$lnRR=\ln\left(\frac{{\bar{X}}_{induction}}{{\bar{X}}_{control}}\right)$$

where ${\bar{X}}_{induction}$ and ${\bar{X}}_{control}$ represent the mean tannin concentrations for induced and control groups, respectively. Corresponding variances of ln*RR* were computed using standard meta-analytic procedures described by Hedges et al. (1999). If multiple tannin groups (total, hydrolysable, or condensed tannins) or multiple measurement time points were reported, each group or time point was treated as a separate effect size, and remaining non-independence among effect sizes was addressed using multilevel random-effects structures. Consequently, a negative ln*RR* value indicates a decrease in leaf tannin concentrations following damage, whereas a positive value indicates an increase (i.e., induction).

The sampling variance of ln*RR* was calculated using the following equation:

$$v_{i}=\left(\frac{{SD}_{induction}^{2}}{n_{induction}{\bar{X}}_{induction}^{2}}\right)+\left(\frac{{SD}_{\text{control}}^{2}}{n_{\text{control}}{\bar{X}}_{\text{control}}^{2}}\right)$$

where *SD* represents the standard deviation, and *n* represents the sample size for each group (Borenstein, 2009).

We used ln*RR* because it provides an intuitive, scale-free measure of proportional change in tannin concentration relative to the control (pre-damage) baseline, facilitating comparison across studies that differ in assays and reporting units. ln*RR* is widely used in ecological meta-analyses of response ratios and has a well-established sampling-variance estimator for inverse-variance weighting (Hedges et al., 1999; Borenstein, 2009).

### 2.4 *Statistical analyses*

All analyses were conducted in R (v.4.4.3) using the *metafor* package (Viechtbauer, 2010). Meta-analytic models were fitted with restricted maximum likelihood (REML), and effects were considered statistically significant when the 95% confidence interval excluded zero.

#### 2.4.1 Phylogenetic signal analysis

We constructed a phylogenetic tree with estimated branch lengths for all 78 species using the *V.PhyloMaker* package (Jin and Qian, 2019) to test whether tannin inducibility estimates (ln*RR*) exhibit phylogenetic signal. Species names were standardized to binomial nomenclature and matched to the phylogeny (final matched set: n = 78 species spanning 42 genera and 30 families; woody = 57; non-woody = 21). Because some species contributed multiple effect sizes, we derived a single species-level estimate of inducibility by computing an inverse-variance weighted mean of ln*RR* within species (*w*_*i*_ = 1/*v*_*i*_), yielding one value per tip. We also repeated the species-level aggregation using arithmetic (unweighted) means as a sensitivity check. We then quantified phylogenetic signal in species-level ln*RR* by estimating Pagel's λ via maximum likelihood and using a likelihood ratio test comparing the fitted model to a model with λ fixed at 0 (*phytools*; Revell, 2012). As a sensitivity analysis to avoid pseudo-replication, we repeated λ estimation using stratified resampling (1000 iterations), selecting one effect size per species in each iteration and summarizing the distribution of λ across iterations. Finally, we repeated the same procedure separately for woody and non-woody plants to evaluate whether woodiness modifies the phylogenetic signal in tannin induction. Importantly, apparent clustering of woody versus non-woody taxa in the phylogeny should not be interpreted as evidence for phylogenetic signal in ln*RR*, because it may reflect phylogenetic conservatism of growth form and uneven taxonomic sampling. Given uneven taxonomic coverage and uncertainty in the reconstructed phylogeny for some taxa, λ estimates were interpreted cautiously.

#### 2.4.2 Meta-analysis

We fitted multilevel meta-analytic models using restricted maximum likelihood (REML) in the *metafor* package, with woodiness (woody vs. non-woody) as the focal moderator. To account for non-independence among effect sizes, we included random intercepts for publications, for effect IDs nested within publications, and for species, with species crossed across publications. We extracted the variance components (*τ*^2^) to quantify and partition heterogeneity across publication, within publication, and species levels, and we report each component as a proportion of the sum of *τ*^2^ across levels. We first fitted an intercept-only model to estimate the overall weighted mean ln*RR* and then added woodiness to test for differences between woody and non-woody plants. We next evaluated each additional moderator in two stages: (i) a model including the moderator as a fixed effect, and (ii) a model including woodiness, the moderator, and their interaction, allowing woody versus non-woody contrasts to be estimated within each moderator level. Moderators included life span (annual plants vs. perennial plants), tannin group (total tannins, hydrolysable tannins, and condensed tannins), leaf damage status (mixed leaves, damaged leaves, and undamaged leaves on damaged plants), leaf cohort (mixed leaves, preexisting leaves, and regrowth leaves), and induction method (mechanical induction, herbivore induction, chemical induction, or joint induction). Residual heterogeneity was quantified and pairwise contrasts among levels within each moderator were evaluated using Wald tests. Effects were considered statistically significant when the 95% confidence interval excluded zero. As a robustness check, we repeated moderator models separately within woody-only and non-woody-only subsets rather than relying solely on interaction terms in the combined model.

We conducted an exploratory analysis of induction regime using ordinary least-squares linear regressions relating ln*RR* to damage intensity, damage duration, and sampling lag. Regressions were fitted for the full dataset and separately for woody and non-woody subsets. For damage intensity, we fitted a no-intercept model (forced through the origin) to reflect the expectation that tannin induction approaches zero as damage approaches zero.

#### 2.4.3 Detection of publication bias

We assessed potential publication and reporting biases using complementary diagnostics commonly applied in ecological meta-analyses (Jennions and Møller, 2002a; Holman et al., 2015). We used Egger's regression test to assess funnel plot asymmetry and visually inspected funnel plots (Egger et al., 1997). We tested for time-lag bias by examining the association between effect size and publication year (Murtaugh, 2002). Furthermore, we explored potential sources of publication bias by examining the correlation between effect sizes and journal impact factors (Jennions and Møller, 2002a), given that null or contradictory findings are often more difficult to publish.

## 3 Results

**Fig. 1:**
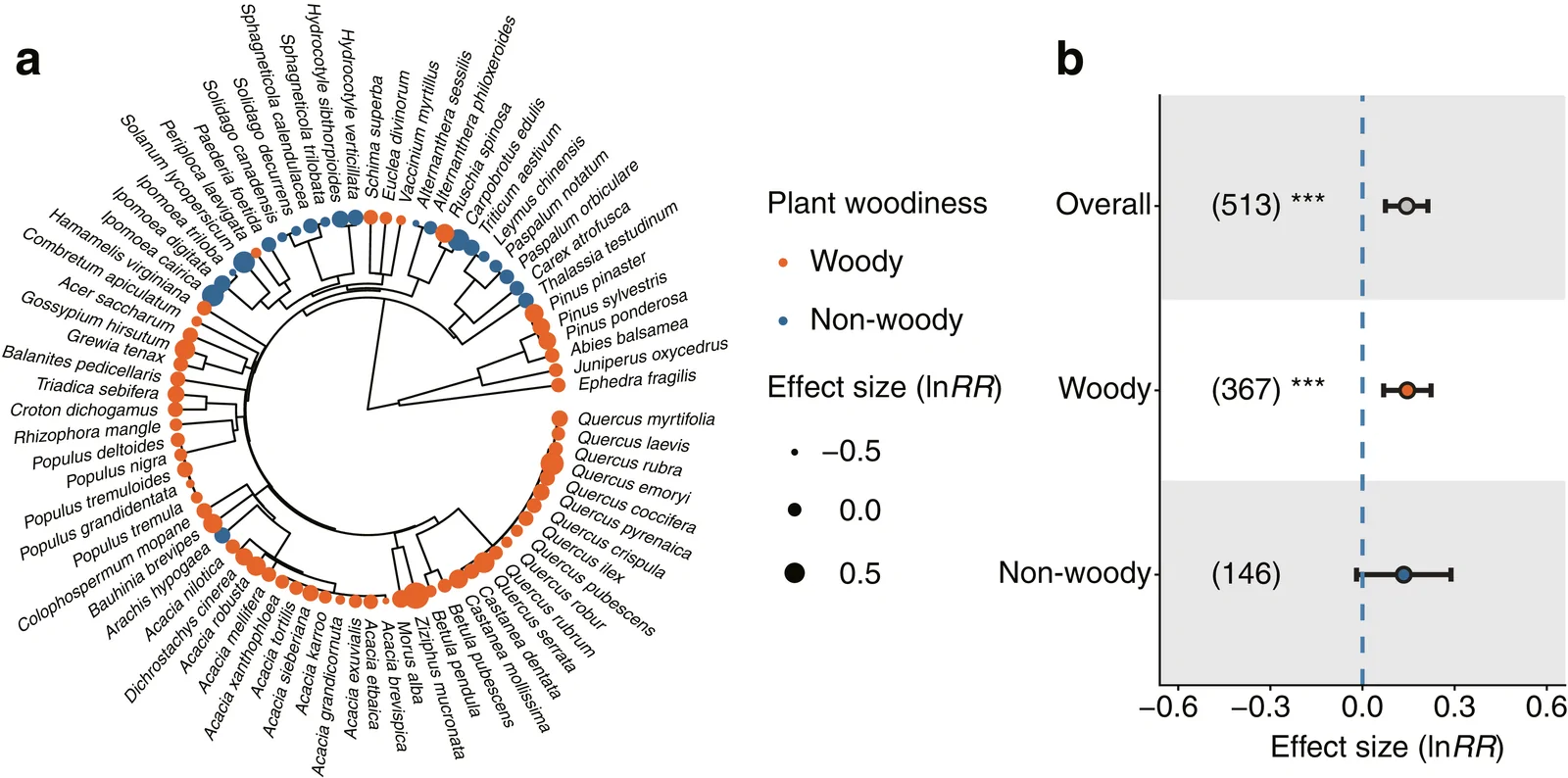
**Phylogenetic distribution and meta-analytic estimates of induced leaf tannin responses. (a)** Circular phylogeny of the 78 plant species included in the analysis, with tips colored by woodiness (woody vs. non-woody). Tip-point size scales with the inverse-variance weighted species mean ln*RR* (*w*_*i*_ = 1/*v*_*i*_). **(b)** Multilevel REML meta-analytic estimates of effect sizes. Overall estimate is from an intercept-only multilevel model, whereas the woody and non-woody estimates are from an otherwise identical model that includes woodiness as a categorical moderator. Points show model-estimated means and horizontal lines denote 95% confidence intervals. The dashed vertical line marks ln*RR* = 0; confidence intervals entirely above zero indicate significantly positive induction, whereas intervals overlapping zero indicate no detectable effect. Numbers in parentheses are the number of effect sizes contributing to each estimate, and asterisks denote statistical significance (∗∗∗*p* < 0.001).

In total, we obtained 513 effect sizes from 78 plant species (57 woody and 21 non-woody), comprising 367 effect sizes from woody taxa and 146 from non-woody taxa. Although non-woody taxa appear clustered in the phylogeny (Fig. 1a), this visual pattern should not be interpreted as evidence of phylogenetic signal in ln*RR*; it likely reflects phylogenetic conservatism of growth form and uneven taxonomic coverage. Using species-level summaries (one value per tip), Pagel's λ was not significantly different from zero for the full dataset (inverse-variance weighted species means; λ = 0.415, *p* = 0.219; arithmetic species means: λ = 0.549, p = 0.069; Table S2). Consistent with these estimates, stratified resampling (one effect size randomly selected per species per iteration; 1000 iterations) produced a low to moderate median λ with substantial uncertainty (median λ = 0.234; 2.5–97.5% quantiles = 0.000–0.821), and λ was significantly different from zero in only 16.9% of iterations (Table S3). A multilevel random-effects model fitted with REML estimated a significantly positive overall weighted effect (ln*RR*; 95% CI that excluded zero; Fig. 1b; Table S4). Heterogeneity among effect sizes was substantial (total *I*^2^ = 93.6%). Variance-component estimates indicated that heterogeneity occurred primarily among studies/publications (*τ*^2^ = 0.047; 52.1%), followed by within-study variation (*τ*^2^ = 0.029; 31.7%) and species-level variation (*τ*^2^ = 0.015; 16.2%; Table S4), with percentages indicating the proportion of the total *τ*^2^. Overall, inducible tannin responses were significantly positive but highly heterogeneous, with weak phylogenetic signal and most variance attributable to between-study differences.

### 3.1 Woody plants versus non-woody plants

Stratified resampling indicated that phylogenetic signal estimates for ln*RR* may differ between woody and non-woody taxa, but the estimates were generally weak and highly uncertain (Table S2; Table S3). For woody species, λ was moderate on average across resamples (median λ = 0.561; 2.5–97.5% quantiles = 0.000–0.842) but reached statistical significance in only 24.4% of iterations (Table S3). In contrast, non-woody species showed λ estimates centered near zero (median λ = 0.000) but with a broad upper tail (2.5–97.5% quantiles = 0.000–0.729), and statistical significance was rare (1.8% of iterations; Table S3). Consistent with these resampling results, species-level analyses based on inverse-variance weighted means indicated no clear departure from λ = 0 for woody plants (λ = 0.410, *p* = 0.263) and an effectively zero estimate for non-woody plants (λ = 0.000, *p* = 1.000; Table S2). Because the non-woody subset included only 21 species and effect sizes were highly heterogeneous, phylogenetic signal estimates for non-woody plants remain imprecise; therefore, the apparent clustering of non-woody taxa in Fig. 1a should not be interpreted as evidence of phylogenetic signal in ln*RR*. In a multilevel random-effects model fitted with REML that estimated separate means for each plant type, tannin induction was significantly positive in woody plants, whereas the non-woody estimate was positive but not significantly different from zero (Fig. 1b; Table S4). Heterogeneity remained high in woodiness-stratified models (Table S4). Life span analyses further indicated significantly positive induction in perennial plants overall and within both woody and non-woody subsets, whereas annual plants showed no detectable induction (annual woody observations were not available; Fig. S1; Table S4). Overall, induction was detectably positive in woody plants, whereas responses in non-woody plants were less consistent and more uncertain, and phylogenetic signal in ln*RR* was generally weak and inconsistent across both species-level and resampling-based assessments (Table S2–S3).

### 3.2 Effects of leaf tannin group, leaf damage status, and leaf cohort

**Fig. 2:**
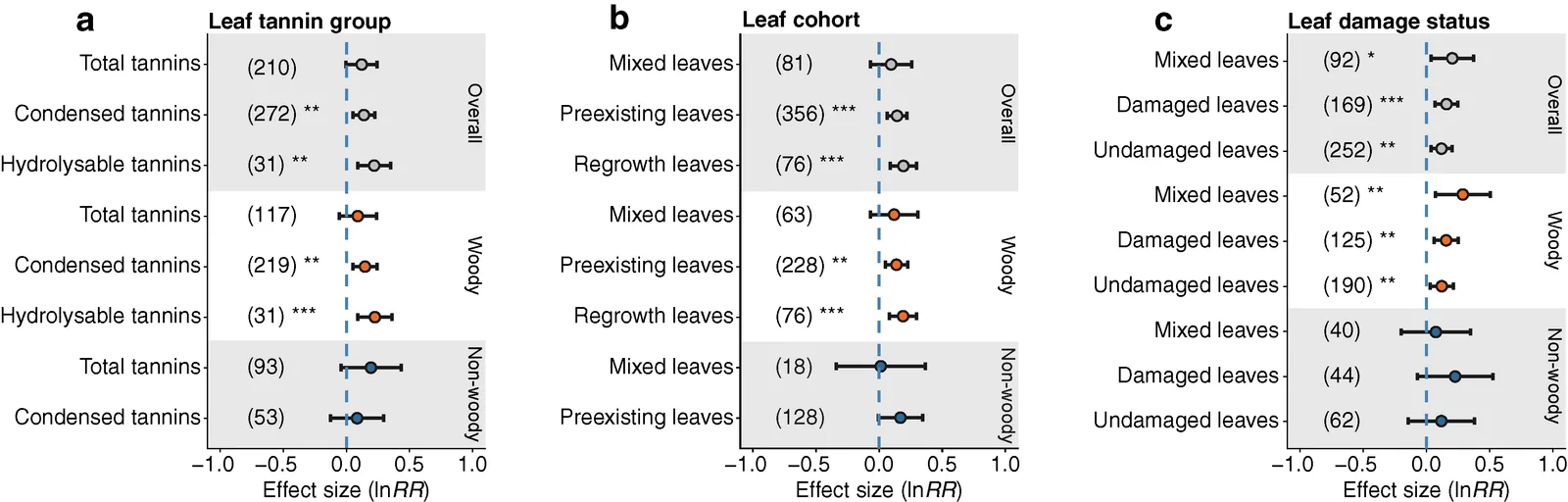
**Categorical moderators of induced leaf tannin responses.** Panels show multilevel REML estimates of effect size (ln*RR*) with 95% confidence intervals (horizontal lines) for **(a)** leaf tannin group, **(b)** leaf cohort, and **(c)** leaf damage status. Total (overall) estimates are from models including the focal moderator only, whereas woody and non-woody estimates are from the corresponding models that additionally include the moderator × woodiness interaction, yielding woodiness-specific means within each moderator level. The dashed vertical line marks ln*RR* = 0; confidence intervals entirely above zero indicate significantly positive induction, whereas intervals overlapping zero indicate no detectable effect. Numbers in parentheses denote the number of effect sizes contributing to each estimate, and asterisks indicate statistical significance (∗*p* < 0.05, ∗∗*p* < 0.01, ∗∗∗*p* < 0.001). Some subgroup estimates were not estimable because of insufficient data (e.g., hydrolysable tannins and regrowth leaves in non-woody plants).

The REML model testing for leaf tannin group as a fixed effect indicated that condensed and hydrolysable tannins both showed significant induced responses, whereas the mean effect size was not significant for total tannins (Fig. 2a; Table S4). In a model including the interaction between tannin group and woodiness, woody plants exhibited significant induction for condensed and hydrolysable tannins but not for total tannins, whereas non-woody plants showed no significant induction in any group (Fig. 2a; Table S4). With leaf damage status as a fixed effect, the REML model indicated significant induction for mixed, damaged, and undamaged leaves on damaged plants (Fig. 2c; Table S4). When the interaction with woodiness was included, woody plants exhibited significant induction, whereas non-woody plants showed no significant induction for any damage-status categories (Fig. 2c; Table S4). With leaf cohort as a fixed effect, the REML model showed significant induction in preexisting and regrowth leaves, whereas mixed leaves were not significant (Fig. 2b; Table S4). Adding the interaction with woodiness produced the same pattern for woody species: preexisting and regrowth leaves were significantly induced and mixed cohorts were not; non-woody species showed no significant induction in any cohort (Fig. 2b; Table S4). No contrasts were significant among leaf tannin groups, leaf damage statuses or leaf cohorts, nor between woody and non-woody plants (Table S5). Subgroup analyses were broadly directionally consistent, though significance sometimes differed due to subset-specific variance structures (Table S7). Overall, woody plants showed consistently positive estimates of inducibility across leaf tannin groups, leaf damage statuses, and leaf cohorts, whereas non-woody plants showed less consistent inducibility, and most formal contrasts among moderator levels and between woodiness categories were non-significant.

### 3.3 Effects of damage intensity, damage duration, and sampling lag

**Fig. 3:**
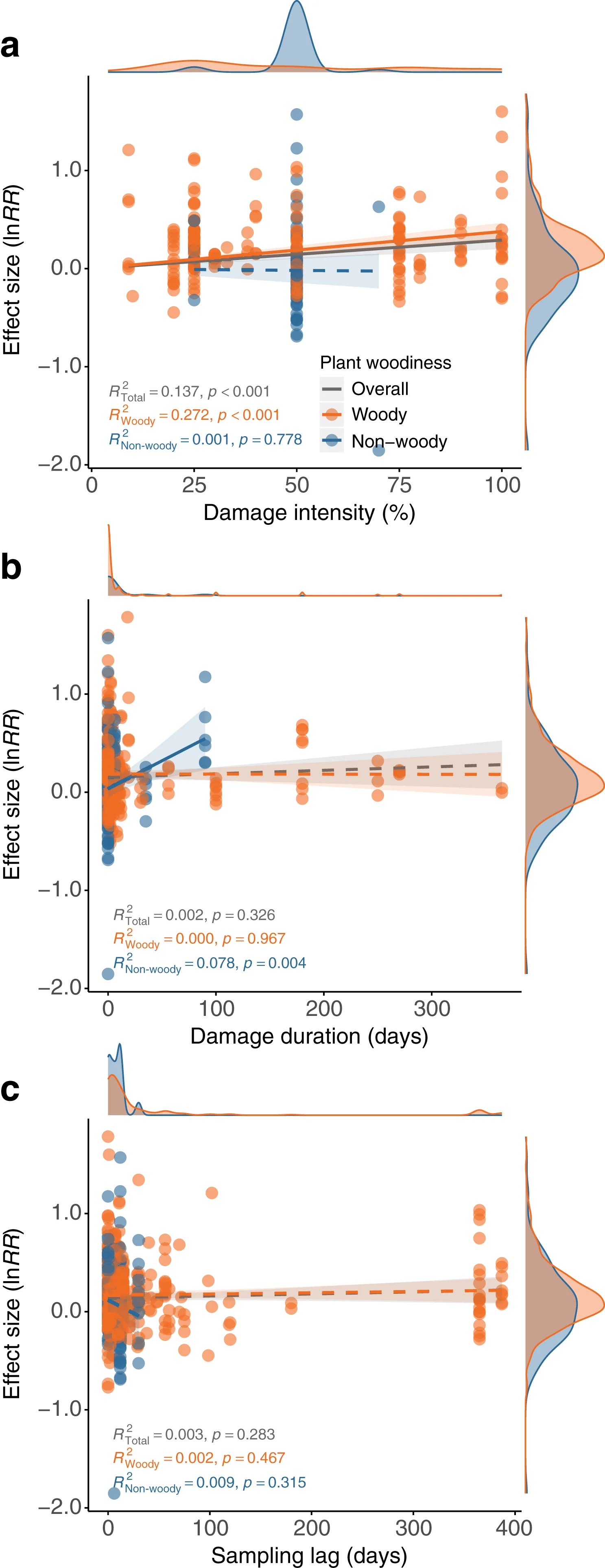
**Effect sizes (ln*RR*) of induced leaf tannin responses plotted against induction regime.** Orange points represent woody plants, and blue points represent non-woody plants. Regression lines are shown for woody plants, non-woody plants, and the pooled dataset (gray), with shaded areas indicating the 95% confidence intervals. Non-significant fits are shown with dashed lines. The marginal density plots on each axis illustrate the distributions of ln*RR* (vertical) and the respective explanatory variable (horizontal). Reported *R*^2^ values and p-values reflect the fit and significance of each linear model.

The relationship between damage intensity and the tannin induction effect size was overall significant (Fig. 3a; Table S6). When analyzing woody and non-woody plants separately, we found a significant relationship between damage intensity and the induction effect size in the case of woody plants, but not in non-woody plants (Fig. 3a; Table S6). The marginal density for non-woody plants reflects the distribution of available observations, which is concentrated at intermediate damage intensities and sparse at the extremes; accordingly, this visual pattern should be interpreted cautiously given the non-significant slope (Table S6). In contrast, the relationship between damage duration and the magnitude of tannin induction was overall not significant (Fig. 3b; Table S6). When analyzing woody and non-woody plants separately, this relationship remained non-significant for woody plants but was significant for non-woody plants (Fig. 3b; Table S6). Lastly, there were no significant relationships between sampling lag and tannin induction effect sizes, either overall or within each woodiness category (Fig. 3c; Table S6). Overall, ln*RR* increased with damage intensity primarily in woody plants, damage duration showed a positive association only in non-woody plants, and sampling lag showed no detectable relationship with ln*RR*.

### 3.4 Effects of induction method

**Fig. 4:**
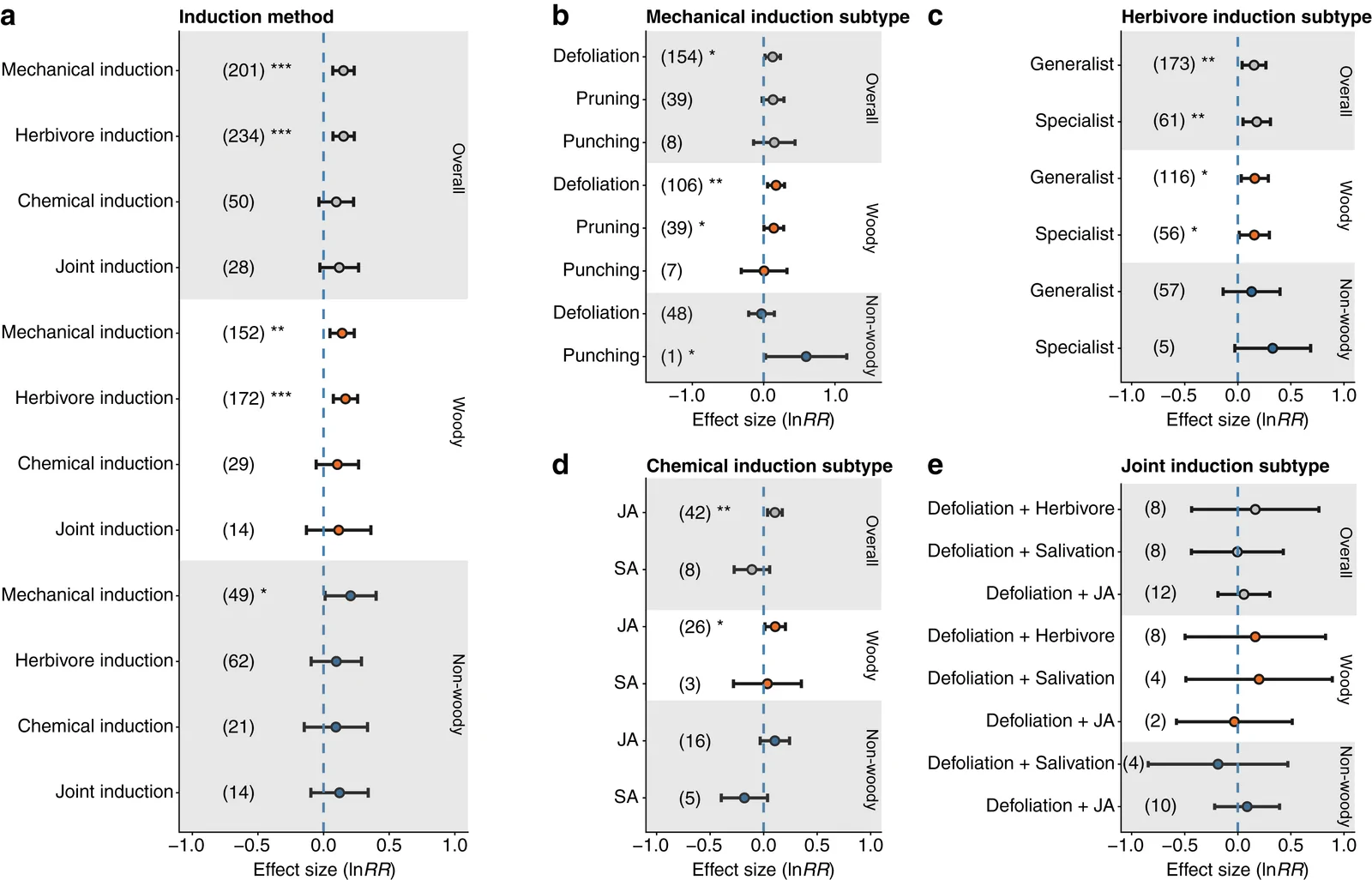
**Induction methods and subtypes as categorical moderators of induced leaf tannin responses.** Panels show multilevel REML estimates of effect size (ln*RR*) with 95% confidence intervals (horizontal lines) for **(a)** induction method (mechanical, herbivore, chemical, joint), **(b)** mechanical induction subtype (defoliation, pruning, punching), **(c)** herbivore induction subtype (i.e., herbivore diet breadth; generalist vs. specialist), **(d)** chemical induction subtype (i.e., chemical elicitor; jasmonic acid [JA] vs. salicylic acid [SA]), and **(e)** joint induction subtype (defoliation + herbivore, defoliation + salivation, and defoliation + JA). Total (overall) estimates are from models including the focal moderator only, whereas woody and non-woody estimates are from the corresponding models that additionally include the moderator × woodiness interaction, yielding woodiness-specific means within each moderator level. The dashed vertical line marks ln*RR* = 0; confidence intervals entirely above zero indicate significantly positive induction, whereas intervals overlapping zero indicate no detectable effect. Numbers in parentheses denote the number of effect sizes contributing to each estimate, and asterisks indicate statistical significance (∗*p* < 0.05, ∗∗*p* < 0.01, ∗∗∗*p* < 0.001). Some subgroup estimates were not estimable because of insufficient data.

The REML models testing induction method type indicated that mechanical and herbivore induction yielded significant induced responses, whereas chemical and joint induction did not differ significantly from zero (Fig. 4a; Table S4). Pairwise contrasts among induction methods were non-significant (Table S5). Including the woodiness × induction method interaction showed that woody plants exhibited significant induction under mechanical and herbivore induction, while chemical and joint induction were non-significant; in non-woody species, only mechanical induction was significant (Fig. 4a; Table S4). Within each induction type, woodiness contrasts were non-significant (Table S5).

Partitioning mechanical damage into subtypes showed significant induction under defoliation, whereas pruning and punching were non-significant (Fig. 4b; Table S4). When examined by woodiness, defoliation induced significant responses in woody plants, and pruning was also significant, whereas punching did not; in non-woody plants, punching reached significance but was represented by a single effect size and should be interpreted cautiously (Fig. 4b; Table S4). Contrasts among mechanical subtypes and contrasts between woody and non-woody plants within subtypes were not significant (Table S5). Similarly, subdividing natural herbivory by herbivore diet breadth showed significant induction by both generalist and specialist herbivores; when examined by woodiness, woody species showed significant induction for both categories, whereas effects for non-woody species were non-significant (Fig. 4c; Table S4). Contrasts between herbivore types and contrasts between woody and non-woody plants within each category were not significant (Table S5). For chemical induction, jasmonic acid (JA) elicited a significant overall induction, whereas salicylic acid (SA) did not; the JA versus SA contrast was significant, but contrasts between woody and non-woody plants within each elicitor were not (Fig. 4d; Table S4; Table S5). Joint induction was non-significant overall and within woody and non-woody plants; differences among the three joint subtypes were also non-significant (Fig. 4e; Table S4; Table S5). Subgroup analyses fitted separately for woody and non-woody datasets yielded moderator patterns that were consistent with those from the corresponding moderator × woodiness interaction models (Table S7). Overall, induction was most consistently detected under mechanical damage and herbivory, particularly defoliation, whereas chemical and joint induction produced weaker and more variable responses and between-woodiness contrasts were generally non-significant. Estimates for sparsely represented strata (*k* < 10) should be interpreted cautiously, as limited sample size reduces precision and robustness.

### 3.5 Publication biases

The funnel plot displayed a generally symmetrical distribution of standard errors across effect sizes, suggesting no strong evidence of small-study effects, which was further confirmed by Egger's regression test indicating no funnel asymmetry (Fig. S2a). We also found no significant relationship between the effect sizes (ln*RR*) and the impact factors of the journals in which the studies were published (Fig. S2b). Additionally, there was a significant relationship between the effect sizes (ln*RR*) and the year of publication (Fig. S2c). Overall, these diagnostics suggest that small-study effects are unlikely to dominate the dataset, but the magnitude of inducibility should be interpreted cautiously given the temporal trend.

## 4 Discussion

Across diverse experimental contexts, we found that woody taxa showed directionally consistent and often detectable positive induction across multiple design strata, whereas estimates for non-woody taxa were more variable and less consistently detectable. Because our meta-analysis quantifies induction rather than realized resistance, the pattern we report for woody taxa should be interpreted as evidence of consistently detectable tannin inducibility, not as evidence of defensive efficacy across all systems. Our phylogenetic analyses detected limited evidence of phylogenetic signal in tannin inducibility within either woody or non-woody taxa. Although weak or non-significant Pagel's λ cannot exclude phylogenetic effects, our ability to detect phylogenetic signal is constrained by uneven taxonomic coverage and uncertainty in the reconstructed tree (Freckleton et al., 2002). Nevertheless, woody taxa showed directionally consistent induction responses across contexts. This pattern is more consistent with shared ecological filters and experimental designs shaping inducibility across lineages than with inducibility being driven primarily by common ancestry (Agrawal, 2007).

Woody taxa showed more consistently positive induction of leaf tannins than non-woody taxa, potentially reflecting greater longevity and a greater capacity for defensive storage and systemic signal transmission (Coley et al., 1985; Herms and Mattson, 1992; Eyles et al., 2010). Consistent with this growth form interpretation, tannin induction was detectable in perennial species, including perennial non-woody taxa, whereas annuals did not show a detectable response in our dataset. Although classical apparency theory predicts that chronic herbivory on large, long-lived plants favors constitutive defense, our synthesis still indicates an association between woodiness and heightened inducibility of quantitative tannins (Feeny, 1976; Coley and Barone, 1996). This combination suggests that constitutive investment and inducible up-regulation need not be mutually exclusive, although we do not quantify trade-offs directly (Koricheva et al., 2004). This pattern reflects changes relative to the baseline and therefore captures inducibility rather than absolute defense levels. It is consistent with traits that covary with woodiness, such as greater storage and transport capacity, which may facilitate rapid up-regulation after damage (Agrawal, 1999; Kant et al., 2015). In contrast, many short-lived herbaceous plants may rely more on alternative defenses or tolerance, yielding weaker or more variable tannin induction (Coley and Barone, 1996; Züst and Agrawal, 2017). In addition, because woodiness covaries with plant size and longevity, woody taxa may be more apparent to herbivores, which could increase repeated attack and contribute to the more consistent induction patterns reported here (Smilanich et al., 2016). However, inducibility also likely varies with functional strategies within woody lineages, so divergence in induced defenses cannot be reduced to a simple woody versus non-woody contrast (Agrawal, 1999; Moreira et al., 2014; Kant et al., 2015; Defossez et al., 2018).

Because many strict between-group contrasts were non-significant, we emphasize the stability of effect direction and the detectability of positive induction in woody plants, rather than inferring inherently stronger inducibility in woody plants. Condensed and hydrolysable tannins show more consistently positive induction in woody taxa, whereas estimates for total tannins are more variable overall. This variability may partly reflect differences in how "total tannins" are operationalized across assays and fractions, which can inflate heterogeneity and dilute induction signals (Salminen and Karonen, 2011; Marsh et al., 2020). Woody taxa show positive induction in both damaged leaves and undamaged leaves on damaged plants, consistent with systemic signaling from damaged tissues to distal leaves (Ryan and Moura, 2002; Karban and Baldwin, 2007). Positive induction was also evident in both preexisting and regrowth leaves, indicating detectability across leaf cohorts and potentially reflecting growth-form differences in storage and transport-related traits (Agrawal, 1999; Kant et al., 2015). In non-woody taxa, induction estimates are more variable, and key strata are sparsely represented, making effect direction and uncertainty harder to resolve. This pattern may reflect constraints in fast-growing herbaceous plants, including lower carbon reserves, reduced storage capacity, and stronger trade-offs between constitutive and induced defenses, which could limit repeated up-regulation of quantitative tannins (Herms and Mattson, 1992; Kempel et al., 2011; Hartmann and Trumbore, 2016; Züst and Agrawal, 2017; Agrawal and Hastings, 2019). However, this is only one plausible explanation; across systems and experimental definitions, non-woody taxa may employ qualitative toxins, tolerance, and context-dependent induction, and thus should not be characterized as universally lacking induction.

Our analyses identified a significant positive association between damage intensity and tannin induction, particularly in woody taxa. This pattern is consistent with damage-dependent up-regulation in woody taxa and may reflect greater capacity for sustained investment in chemical defenses (Barbehenn and Constabel, 2011; Constabel et al., 2014). In non-woody taxa, the corresponding relationship was not statistically significant, which may reflect constraints on resource allocation but could also arise from sparse coverage and heterogeneous experimental definitions (Herms and Mattson, 1992; Koricheva et al., 2004). In addition, we detected a significant positive relationship between damage duration (the length of time plants were continuously or repeatedly exposed to damage) and tannin induction only in non-woody taxa. This result suggests that prolonged or repeated damage may be required for induction to be detectable in some rapidly growing non-woody taxa (Clausen et al., 1992; Underwood, 2012). Finally, we found no significant association between sampling lag and tannin induction in the pooled dataset or within woody and non-woody subsets. Rather than implying temporal stability, this non-significant association may reflect heterogeneous sampling windows and timing-dependent induction dynamics across studies (Stevens and Lindroth, 2005; Kant et al., 2015).

Our results suggest that induction method contributes to variation in the detectability and directionality of tannin induction. Across the pooled dataset, ln*RR* estimates were positive for mechanical damage and herbivore induction, whereas estimates for chemical (JA/SA) and joint induction were more heterogeneous, especially in woody taxa. This pattern is consistent with a damage-centered response in which pronounced tissue removal, particularly defoliation, may elicit more consistent up-regulation of quantitative defenses and systemic signaling to distal leaves (Ryan and Moura, 2002; Stevens and Lindroth, 2005; Karban and Baldwin, 2007), as reported for woody systems such as *Populus* (Arnold and Schultz, 2002; Arnold et al., 2004). Within mechanical induction, defoliation produced the most consistently positive estimates. In contrast, pruning and punching were less well resolved, likely reflecting small sample sizes, heterogeneous protocols, and variable sampling windows, and possibly more localized or weaker damage responses. Under herbivore induction, woody taxa showed positive induction with both generalist and specialist herbivores, whereas non-woody estimates were more uncertain and based on sparse evidence, a pattern more consistent with limited power than with a true null effect. Within chemical induction, JA elicitation increased tannins in the pooled dataset and in woody taxa, whereas SA showed no detectable effect. This contrast is broadly consistent with JA-mediated herbivore defense signaling and potential JA and SA crosstalk (Rojo et al., 2003; Thaler et al., 2004). However, because exogenous elicitors are mechanistic probes rather than ecological analogs of intact herbivory, their effects are strongly dose- and timing-dependent and may not translate directly to natural feeding responses (Rojo et al., 2003; Karban and Baldwin, 2007; Constabel et al., 2014). The non-significant pooled effect of joint induction may reflect limited sample size and highly heterogeneous treatment regimes and does not rule out synergistic, antagonistic, or timing-dependent interactions. Moreover, signal stacking need not be additive, because synergy, saturation, antagonism, and timing mismatches can shift effect directions across systems and drive pooled estimates toward zero (Rojo et al., 2003; Karban and Baldwin, 2007).

Publication and reporting biases could influence our conclusions; such biases are well documented in ecological meta-analyses (Jennions and Møller, 2002a; Murtaugh, 2002). Induced-defense studies may be especially vulnerable because responses can be quantified across tissues, chemical fractions, and sampling windows, and because tannin metrics and assays have changed through time, increasing flexibility in what is most readily detected and reported (Salminen and Karonen, 2011; Marsh et al., 2020; Garcia et al., 2021; Ojha et al., 2022). Our diagnostics did not suggest that the overall pattern is dominated by small-study effects or selective publication (Egger et al., 1997). However, these tests have limited power under high heterogeneity and cannot exclude subtler biases, including selective outcome reporting and incomplete reporting of variance and sample sizes that can constrain inclusion in variance-weighted analyses (Nakagawa and Santos, 2012). We also observed temporal variation in effect sizes, which may reflect time-lag processes and gradual shifts in protocols, assays, and reporting conventions over decades (Jennions and Møller, 2002b; Murtaugh, 2002). These considerations warrant caution when interpreting the exact magnitude of inducibility, particularly for sparsely sampled strata. However, they are less likely to overturn our central inference, which is supported by directionally consistent positive inducibility in woody taxa across multiple methodological contrasts rather than by any single subset of studies or assay definition.

Nonetheless, our inferences are limited to tannins, and other defensive metabolite classes may show different inducibility patterns and allocation trade-offs. Residual heterogeneity likely reflects variation in experimental designs and tannin assays, together with uneven taxonomic and categorical coverage that leaves some strata sparsely sampled and limits power for fine-grained contrasts. Future progress would benefit from standardized protocols, complete reporting of sample sizes and variance, and broader phylogenetically informed sampling across growth forms and regions. Critically, woodiness should be embedded within a trait-based life history framework by testing how storage- and transport-related traits, growth rate, and resource acquisition jointly shape inducibility and its trade-offs with constitutive investment across woody and non-woody taxa. Mechanistic experiments that manipulate damage regimes and signaling pathways, ideally linking chemical responses to fitness-related outcomes, would strengthen causal inference. Extending comparable tests to other defense classes (e.g., non-tannin phenolics, terpenoids, and alkaloids) would help determine whether the woodiness-associated pattern observed here is tannin-specific or general across chemical defenses. Together, these results motivate a more predictive, trait-based view of when and where tannin induction is detectable, while highlighting key gaps in taxonomic coverage and experimental standardization.

## CRediT author contributions

**Keke Sun:** Data curation (lead); Formal analysis (lead); Writing – original draft (lead); Writing – review & editing (equal). **Jianwei Wang:** Data curation (equal); Writing – original draft (equal); Writing – review & editing (equal). **Yanling Zhang:** Investigation (lead); Visualization (equal); Writing – original draft (equal). **Youzheng Zhang:** Writing – review & editing (supporting). **Lele Liu:** Writing – review & editing (supporting). **Luis Abdala-Roberts:** Investigation (supporting); Formal analysis (supporting); Writing – review & editing (lead). **Yaolin Guo:** Conceptualization (lead); Investigation (lead); Supervision (lead); Visualization (equal); Writing – original draft (lead); Writing – review & editing (lead).

## Data availability statement

The data supporting this study are archived in the Science Data Bank (ScienceDB) and are available at: https://doi.org/10.57760/sciencedb.j00143.00140. The analysis code is available on GitHub at: https://github.com/yaolinguo/tannin_induction.

## Declaration of competing interest

The authors declare that they have no known competing financial interests or personal relationships that could have appeared to influence the work reported in this paper.
