# Supplementary material for: Heterogeneity in leaf tannin inducibility across woody and non-woody plants: A meta-analysis
Source: Plant Divers. 2026 Feb 24;48(5):986–96. doi: 10.1016/j.pld.2026.02.007 (PMC13615789; doi:10.1016/j.pld.2026.02.007)
Supplement: Supplementary file 1 [file mmc1.pdf]

**Supplementary files**

**This file includes:**

Tables S1 to S7

Figures S1 and S2

**Table S1 Primary studies included in the meta-analysis and associated effect-size records.**

This table lists all publications from which effect sizes were extracted for the meta-analysis of induced plant responses. Individual studies may contribute multiple effect sizes because of differences in species, genotypes/populations, treatments, tissues, or sampling times within the same article.

| No. | Reference (first author and year) | Journal | Article title |
|---|---|---|---|
| 1 | Arnold and Schultz, 2002 | Oecologia | Induced sink strength as a prerequisite for induced tannin biosynthesis in developing leaves of *Populus* |
| 2 | Arnold et al., 2008 | Aquatic Botany | Wound-induced accumulations of condensed tannins in turtlegrass, *Thalassia testudinum* |
| 3 | Arnold et al., 2004 | New Phytologist | Carbohydrate translocation determines the phenolic content of *Populus* foliage: a test of the sink–source model of plant defense |
| 4 | Baraza et al., 2004 | Canadian Journal of Botany | Herbivory has a greater impact in shade than in sun: response of *Quercus pyrenaica* seedlings to multifactorial environmental variation |

| | | | |
|---|---|---|---|
| 5 | Białczyk et al., 1999 | The Journal of Agricultural Science | The protective action of tannins against glasshouse whitefly in tomato seedlings |
| 6 | Boeckler et al., 2013 | Journal of Chemical Ecology | Gypsy moth caterpillar feeding has only a marginal impact on phenolic compounds in old-growth black poplar |
| 7 | Chen et al., 2011 | Acta Oecologica | Defense signaling among interconnected ramets of a rhizomatous clonal plant, induced by jasmonic-acid application |
| 8 | Cooper and Rieske, 2008 | Chemoecology | Differential responses in American (*Castanea dentata* Marshall) and Chinese (*C. mollissima* Blume) chestnut (Falales: Fagaceae) to foliar application of jasmonic acid |
| 9 | Cornelissen and Fernandes, 2001 | Trees | Induced defences in the neotropical tree *Bauhinia brevipes* (Vog.) to herbivory: effects of damage-induced changes on leaf quality and insect attack |
| 10 | Dai et al., 2014 | Israel Journal of Ecology and | Evaluation of the variations in secondary metabolite concentrations |

| | | | |
|---|---|---|---|
| | | Evolution | of *Leymus chinensis* seedlings |
| 11 | Ding et al., 2025 | Arthropod-Plant Interactions | The adaptive ability of *Hyphantria cunea* larvae to induced chemical defenses of *Morus alba*, a high-preference host plant |
| 12 | Eisenring et al., 2021 | Functional Ecology | Spatial, genetic and biotic factors shape within-crown leaf trait variation and herbivore performance in a foundation tree species |
| 13 | Erickson et al., 2004 | Biotropica | Does mangrove leaf chemistry help explain crab herbivory patterns? |
| 14 | Faeth, 1992 | Journal of Chemical Ecology | Do defoliation and subsequent phytochemical responses reduce future herbivory on oak trees? |
| 15 | Filella et al., 2019 | Acta Oecologica | Tolerance to severe browsing of three shrub species on Mediterranean islands |
| 16 | Frost and Hunter, 2008 | Oikos | Insect herbivores and their frass affect *Quercus rubra* leaf quality and initial stages of subsequent litter decomposition |

| | | | |
|---|---|---|---|
| 17 | Frost et al., 2012 | Functional Ecology | A petiole-galling insect herbivore decelerates leaf lamina litter decomposition rates |
| 18 | Fyllas et al., 2022 | Tree Physiology | Photosynthetic and defensive responses of two Mediterranean oaks to insect leaf herbivory |
| 19 | Gallardo et al., 2019 | Physiologia Plantarum | Regulation by biotic stress of tannins biosynthesis in *Quercus ilex*: Crosstalk between defoliation and *Phytophthora cinnamomi* infection |
| 20 | Gaur et al., 2022 | Scientific Reports | Compensatory phenolic induction dynamics in aspen after aphid infestation |
| 21 | Gowda, 2003 | Functional Ecology | Physical and chemical response of juvenile *Acacia tortilis* trees to browsing. Experimental evidence |
| 22 | Gu et al., 2014 | Ecological Entomology | Invasive plant population and herbivore identity affect latex induction |
| 23 | Hean and Ward, 2011 | Journal of Vegetation | Fire and herbivory are not substitutable: evidence from regrowth |

| | | | |
|---|---|---|---|
| | | Science | patterns and changes in physical and chemical defences in *Acacia* seedlings |
| 24 | Holeski et al., 2012 | Oecologia | Relative importance of genetic, ontogenetic, induction, and seasonal variation in producing a multivariate defense phenotype in a foundation tree species |
| 25 | Huang et al., 2013 | Proceedings of the Royal Society B: Biological Sciences | Facilitation and inhibition: changes in plant nitrogen and secondary metabolites mediate interactions between above-ground and below-ground herbivores |
| 26 | Huttunen et al., 2008 | Chemoecology | Does defoliation induce chemical and morphological defenses in the leaves of silver birch seedlings under changing climate? |
| 27 | Kaur et al., 2017 | Journal of Plant Interactions | Role of defense enzymes and phenolics in resistance of wheat crop (*Triticum aestivum* L.) towards aphid complex |
| 28 | Keinänen et al., 1999 | Ecology | Trade-offs in phenolic metabolism of silver birch: effects of fertilization, |

| | | | |
|---|---|---|---|
| | | | defoliation, and genotype |
| 29 | Kohi et al., 2010 | African Journal of Ecology | Effects of simulated browsing on growth and leaf chemical properties in *Colophospermum mopane* saplings |
| 30 | Lauer and Rossi, 2011 | Florida Entomologist | Effects of manual damage on Turkey oak (Fagales: Fagaceae) foliar tannin concentration and subsequent herbivorous insect abundance |
| 31 | Li et al., 2020 | Plant and Soil | Effects of above-and belowground herbivory of specialists and generalists on the growth and defensive chemicals of introduced and native Chinese tallow seedlings |
| 32 | Li et al., 2016 | Oecologia | Plant genotypes affect aboveground and belowground herbivore interactions by changing chemical defense |
| 33 | Lindroth and Kinney, 1998 | Journal of Chemical Ecology | Consequences of enriched atmospheric $CO_2$ and defoliation for foliar chemistry and gypsy moth performance |

| | | | |
|---|---|---|---|
| 34 | Liu et al., 2012 | PLoS One | High tolerance to salinity and herbivory stresses may explain the expansion of *Ipomoea cairica* to salt marshes |
| 35 | Liu et al., 2015 | Pakistan Journal of Botany | Defensive responses of *Poplus deltoides* 895 seedlings against exogenous methyl jasmonate |
| 36 | Liu et al., 2010a | Phyton-Revista Internacional de Botanica Experimental | Induced responses in the subtropical evergreen, broad-leaf tree *Schima superba*: effects of simulated herbivory on leaf quality and subsequent insect attack during leaf expansion |
| 37 | Liu et al., 2010b | African Journal of Biotechnology | Induced response in *Schima superba*: Effects of early-season herbivory on leaf traits and subsequent insect attack |
| 38 | Ma et al., 2018 | Journal of Integrative Agriculture | The influence of *Tetranychus cinnabarinus*-induced plant defense responses on *Aphis gossypii* development |
| 39 | Mizumachi et al., 2011 | Journal of Forest | Variation in herbivory-induced responses within successively flushing |

| | | | |
|---|---|---|---|
| | | Research | *Quercus serrata* seedlings under different nutrient conditions |
| 40 | Monroy and García-Verdugo, 2019 | American Journal of Botany | Testing the hypothesis of loss of defenses on islands across a wide latitudinal gradient of *Periploca laevigata* populations |
| 41 | Nabeshima et al., 2001 | Journal of Plant Research | Effects of herbivory and light conditions on induced defense in *Quercus crispula* |
| 42 | Nosko and Embury, 2018 | Plant Ecology | Induction and persistence of allelochemicals in the foliage of balsam fir seedlings following simulated browsing |
| 43 | Nosko et al., 2020 | Forest Ecology and Management | Growth and chemical responses of balsam fir saplings released from intense browsing pressure in the boreal forests of western Newfoundland, Canada |
| 44 | Osier and Lindroth, 2001 | Journal of Chemical Ecology | Effects of genotype, nutrient availability, and defoliation on aspen phytochemistry and insect performance |

| | | | |
|---|---|---|---|
| 45 | Osier and Lindroth, 2004 | Oecologia | Long-term effects of defoliation on quaking aspen in relation to genotype and nutrient availability: plant growth, phytochemistry and insect performance |
| 46 | Peters and Constabel, 2002 | The Plant Journal | Molecular analysis of herbivore-induced condensed tannin synthesis: cloning and expression of dihydroflavonol reductase from trembling aspen (*Populus tremuloides*) |
| 47 | Portela et al., 2019 | Journal of Plant Ecology | Effects of physiological integration on defense strategies against herbivory by the clonal plant *Alternanthera philoxeroides* |
| 48 | Quijano-Medina et al., 2021 | Journal of Ecology | Effects of soil salinity on the expression of direct and indirect defences in wild cotton *Gossypium hirsutum* |
| 49 | Rehill and Schultz, 2012 | Journal of Chemical Ecology | *Hormaphis hamamelidis* fundatrices benefit by manipulating phenolic metabolism of their host |

| | | | |
|---|---|---|---|
| 50 | Rodríguez et al., 2023 | Biological Invasions | Variation in defensive traits against herbivores of native and invasive populations of *Carpobrotus edulis* |
| 51 | Roitto et al., 2009 | Tree Physiology | Induced accumulation of phenolics and sawfly performance in Scots pine in response to previous defoliation |
| 52 | Rooke and Bergström, 2007 | Plant Ecology | Growth, chemical responses and herbivory after simulated leaf browsing in *Combretum apiculatum* |
| 53 | Rossi et al., 2004 | Journal of Chemical Ecology | Induced defensive response of myrtle oak to foliar insect herbivory in ambient and elevated $CO_2$ |
| 54 | Rubert-Nason et al., 2015 | Journal of Chemical Ecology | Influence of genotype, environment, and gypsy moth herbivory on local and systemic chemical defenses in trembling aspen (*Populus tremuloides*) |
| 55 | Ruohomaki et al., 1996 | Ecology | Delayed inducible resistance in mountain birch in response to fertilization and shade |
| 56 | Sampedro et al., 2011 | Journal of Ecology | Costs of constitutive and herbivore-induced chemical defences in pine |

| | | | |
|---|---|---|---|
| | | | trees emerge only under low nutrient availability |
| 57 | Schultz and Baldwin, 1982 | Science | Oak leaf quality declines in response to defoliation by gypsy moth larvae |
| 58 | Scogings et al., 2014 | Plant Ecology | Nutrient and secondary metabolite concentrations in a savanna are independently affected by large herbivores and shoot growth rate |
| 59 | Sebata and Ndlovu, 2015 | African Journal of Ecology | Within-plant variation in defences in response to simulated herbivory in a semi-arid southern African savannah |
| 60 | Shan et al., 2024 | Ecological Applications | Nutrient enrichment promotes invasion success of alien plants via increased growth and suppression of chemical defenses |
| 61 | Stevens and Lindroth, 2005 | Oecologia | Induced resistance in the indeterminate growth of aspen (*Populus tremuloides*) |
| 62 | Stevens et al., 2012 | Journal of Chemical Ecology | Genotypic differences and prior defoliation affect re-growth and phytochemistry after coppicing in |

| | | | |
|---|---|---|---|
| | | | *Populus tremuloides* |
| 63 | Stevens et al., 2007 | Evolutionary Ecology | Resistance and tolerance in *Populus tremuloides*: genetic variation, costs, and environmental dependency |
| 64 | Stock et al., 1993 | Oecologia | Regrowth and tannin production in woody and succulent karoo shrubs in response to simulated browsing |
| 65 | Strengbom et al., 2003 | Oikos | Effects of repeated damage and fertilization on palatability of *Vaccinium myrtillus* to grey sided voles, *Clethrionomys rufocanus* |
| 66 | Visakorpi et al., 2020 | PLoS One | Changes in oak (*Quercus robur*) photosynthesis after winter moth (*Operophtera brumata*) herbivory are not explained by changes in chemical or structural leaf traits |
| 67 | Wagner and Evans, 1985 | Oecologia | Defoliation increases nutritional quality and allelochemics of pine seedlings |
| 68 | Wang et al., 2012 | Journal of Ecology | Genetic variation in anti-herbivore chemical defences in an invasive plant |

| | | | |
|---|---|---|---|
| 69 | Wang et al., 2016 | BioControl | Repeated damage by specialist insects suppresses the growth of a high tolerance invasive tree |
| 70 | War et al., 2015 | Pest Management Science | Induced resistance to *Helicoverpa armigera* through exogenous application of jasmonic acid and salicylic acid in groundnut, *Arachis hypogaea* |
| 71 | War et al., 2013 | Journal of Plant Growth Regulation | Defensive responses in groundnut against chewing and sap-sucking insects |
| 72 | Wigley et al., 2022 | Austral Ecology | No evidence for the simultaneous induction of structural and chemical defences in spiny southern African savanna trees |
| 73 | Xiao et al., 2019 | AoB Plants | Herbivore-specific induction of indirect and direct defensive responses in leaves and roots |

11

**Table S2 Phylogenetic signal (Pagel's λ) in tannin-induction effect sizes (ln*RR*) estimated at the species level.** Multiple effect sizes within each species were aggregated to a single species value using either the arithmetic mean or an inverse-variance weighted mean. Pagel's λ was estimated by maximum likelihood and tested against $\lambda = 0$ using a likelihood ratio test. Shown are λ, log-likelihood (logL), the test *p*-value, and the number of species/tips included. Group-specific estimates for woody and non-woody species are based on inverse-variance weighted species means.

| Group | Aggregation | Species (n) | Tips (n) | lambda | logL | *P* |
|---|---|---|---|---|---|---|
| All | Species-level mean | 78 | 78 | 0.549 | -7.638 | 0.069 |
| All | Inverse-variance weighted | 78 | 78 | 0.415 | -8.538 | 0.219 |
| Woody | Inverse-variance weighted | 57 | 57 | 0.410 | -2.983 | 0.263 |
| Non-woody | Inverse-variance weighted | 21 | 21 | 0.000 | -5.000 | 1.000 |

**Table S3 Phylogenetic signal (Pagel's λ) in tannin induction effect sizes (ln*RR*) based on stratified resampling.** Values summarize 1,000 iterations in which one effect size was randomly selected per species per iteration. Reported are the median λ and its 2.5% and 97.5% quantiles across iterations, the proportion of iterations with λ significantly different from zero ($p < 0.05$), and the median number of species tips included in each analysis.

| Group | Iterations (n) | Valid (n) | λ (median) | λ (2.5%) | λ (97.5%) | $p < 0.05$ (proportion) | Tips (n) |
|---|---|---|---|---|---|---|---|
| All | 1000 | 1000 | 0.234 | 0.000 | 0.821 | 0.169 | 78 |
| Woody | 1000 | 1000 | 0.561 | 0.000 | 0.842 | 0.244 | 57 |
| Non-woody | 1000 | 1000 | 0.000 | 0.000 | 0.729 | 0.018 | 21 |

**Table S4 Restricted maximum-likelihood (REML) multilevel meta-analytic models testing each moderator and moderator × woodiness interactions.** Moderator variables with 95% confidence intervals (CIs) that do not cross zero indicate statistical significance. LCI = 95% lower CI, UCI = 95% upper CI. Effect sizes (ln*RR*) are the response variable of the REML model and the estimate represents the ln*RR* estimated value for the corresponding variable in the model. Variance components: $\tau_1^2$ = between-study variance (random intercept for publication ID), $\tau_2^2$ = within-study variance among effect sizes (random intercept for effect ID nested within publication), and $\tau_3^2$ = between-species variance (random intercept for species ID, cross-classified across studies). $Q_E$ = test statistic for residual heterogeneity (Cochran's Q) for the fitted model.

| Moderator variables | Estimate | *SE* | *z* | *p* | LCI | UCI |
|---|---|---|---|---|---|---|
| Model 1: Effect size (ln*RR*) ~ 1 | | | | | | |
| ($\tau_1^2 = 0.047$, $\tau_2^2 = 0.029$, $\tau_3^2 = 0.015$; $Q_E = 7972.115$, $df = 512$, $p < 0.001$) | | | | | | |
| **Full model** | **0.144** | **0.035** | **4.120** | **< 0.001** | **0.076** | **0.213** |
| Model 2: Effect size (ln*RR*) ~ Woodiness | | | | | | |
| ($\tau_1^2 = 0.048$, $\tau_2^2 = 0.029$, $\tau_3^2 = 0.015$; $Q_E = 7942.753$, $df = 511$, $p < 0.001$) | | | | | | |
| **Woody** | **0.146** | **0.039** | **3.75** | **< 0.001** | **0.070** | **0.223** |
| Non-woody | 0.135 | 0.078 | 1.730 | 0.084 | -0.018 | 0.288 |
| Model 3: Effect size (ln*RR*) ~ Leaf tannin group | | | | | | |
| ($\tau_1^2 = 0.048$, $\tau_2^2 = 0.028$, $\tau_3^2 = 0.016$; $Q_E = 7272.348$, $df = 510$, $p < 0.001$) | | | | | | |

| | | | | | | |
|---|---|---|---|---|---|---|
| Total tannins | 0.123 | 0.063 | 1.960 | 0.050 | 0.000 | 0.245 |
| **Condensed tannins** | **0.139** | **0.044** | **3.186** | **0.001** | **0.053** | **0.224** |
| **Hydrolysable tannins** | **0.220** | **0.067** | **3.295** | **< 0.001** | **0.089** | **0.351** |
| Model 4: Effect size (ln*RR*) ~ Leaf tannin group × Woodiness | | | | | | |
| ($\tau_1^2 = 0.049$, $\tau_2^2 = 0.028$, $\tau_3^2 = 0.018$; $Q_E = 6579.902$, $df = 508$, $p < 0.001$) | | | | | | |
| Total tannins – Woody | 0.092 | 0.075 | 1.229 | 0.219 | -0.055 | 0.239 |
| **Condensed tannins – Woody** | **0.148** | **0.048** | **3.100** | **0.002** | **0.054** | **0.241** |
| **Hydrolysable tannins – Woody** | **0.226** | **0.068** | **3.310** | **< 0.001** | **0.092** | **0.360** |
| Total tannins – Non-woody | 0.196 | 0.121 | 1.619 | 0.105 | -0.041 | 0.433 |
| Condensed tannins – Non-woody | 0.086 | 0.107 | 0.802 | 0.423 | -0.124 | 0.295 |
| Hydrolysable tannins – Non-woody | NA | NA | NA | NA | NA | NA |
| Model 5: Effect size (ln*RR*) ~ Leaf damage status | | | | | | |
| ($\tau_1^2 = 0.049$, $\tau_2^2 = 0.029$, $\tau_3^2 = 0.015$; $Q_E = 7656.594$, $df = 510$, $p < 0.001$) | | | | | | |
| **Mixed leaves** | **0.201** | **0.084** | **2.407** | **0.016** | **0.037** | **0.366** |
| **Damaged leaves** | **0.156** | **0.045** | **3.494** | **< 0.001** | **0.069** | **0.244** |
| **Undamaged leaves** | **0.118** | **0.042** | **2.816** | **0.005** | **0.036** | **0.200** |

| | | | | | | |
|---|---|---|---|---|---|---|
| Model 6: Effect size (ln*RR*) ~ Leaf damage status × Woodiness | | | | | | |
| ($\tau_1^2 = 0.051$, $\tau_2^2 = 0.029$, $\tau_3^2 = 0.016$; $Q_E = 6344.969$, $df = 507$, $p < 0.001$) | | | | | | |
| **Mixed leaves – Woody** | **0.284** | **0.109** | **2.607** | **0.009** | **0.070** | **0.497** |
| **Damaged leaves – Woody** | **0.154** | **0.048** | **3.197** | **0.001** | **0.059** | **0.248** |
| **Undamaged leaves – Woody** | **0.120** | **0.045** | **2.647** | **0.008** | **0.031** | **0.209** |
| Mixed leaves – Non-woody | 0.074 | 0.136 | 0.541 | 0.588 | -0.194 | 0.341 |
| Damaged leaves – Non-woody | 0.223 | 0.148 | 1.505 | 0.132 | -0.068 | 0.514 |
| Undamaged leaves – Non-woody | 0.118 | 0.131 | 0.897 | 0.370 | -0.140 | 0.375 |
| Model 7: Effect size (ln*RR*) ~ Leaf cohort | | | | | | |
| ($\tau_1^2 = 0.047$, $\tau_2^2 = 0.029$, $\tau_3^2 = 0.016$; $Q_E = 7904.659$, $df = 510$, $p < 0.001$) | | | | | | |
| Mixed leaves | 0.095 | 0.082 | 1.167 | 0.243 | -0.065 | 0.256 |
| **Preexisting leaves** | **0.137** | **0.040** | **3.424** | **< 0.001** | **0.059** | **0.216** |
| **Regrowth leaves** | **0.196** | **0.056** | **3.498** | **< 0.001** | **0.086** | **0.306** |
| Model 8: Effect size (ln*RR*) ~ Leaf cohort × Woodiness | | | | | | |
| ($\tau_1^2 = 0.049$, $\tau_2^2 = 0.029$, $\tau_3^2 = 0.016$; $Q_E = 7740.110$, $df = 508$, $p < 0.001$) | | | | | | |
| Mixed leaves – Woody | 0.119 | 0.094 | 1.268 | 0.205 | -0.065 | 0.303 |

| | | | | | | |
|---|---|---|---|---|---|---|
| **Preexisting leaves – Woody** | **0.131** | **0.045** | **2.902** | **0.004** | **0.043** | **0.220** |
| **Regrowth leaves – Woody** | **0.195** | **0.057** | **3.397** | **< 0.001** | **0.082** | **0.307** |
| Mixed leaves – Non-woody | 0.013 | 0.177 | 0.076 | 0.939 | -0.333 | 0.360 |
| Preexisting leaves – Non-woody | 0.167 | 0.088 | 1.900 | 0.057 | -0.005 | 0.339 |
| Regrowth leaves – Non-woody | NA | NA | NA | NA | NA | NA |
| Model 9: Effect size (ln*RR*) ~ Life span | | | | | | |
| ($\tau_1^2$ = 0.052, $\tau_2^2$ = 0.029, $\tau_3^2$ = 0.012; $Q_E$ = 7537.024, *df* = 511, $p < 0.001$) | | | | | | |
| **Perennial plants** | **0.156** | **0.036** | **4.294** | **< 0.001** | **0.085** | **0.227** |
| Annual plants | 0.015 | 0.118 | 0.130 | 0.897 | -0.215 | 0.246 |
| Model 10: Effect size (ln*RR*) ~ Life span × Woodiness | | | | | | |
| ($\tau_1^2$ = 0.054, $\tau_2^2$ = 0.029, $\tau_3^2$ = 0.012; $Q_E$ = 6659.818, *df* = 510, $p < 0.001$) | | | | | | |
| **Perennial plants – Woody** | **0.150** | **0.039** | **3.815** | **< 0.001** | **0.073** | **0.227** |
| **Perennial plants – Non-woody** | **0.195** | **0.090** | **2.174** | **0.030** | **0.019** | **0.371** |
| Annual plants – Woody | NA | NA | NA | NA | NA | NA |
| Annual plants – Non-woody | 0.021 | 0.120 | 0.177 | 0.859 | -0.215 | 0.257 |
| Model 11: Effect size (ln*RR*) ~ Induction method | | | | | | |

| | | | | | | |
|---|---|---|---|---|---|---|
| ($\tau_1^2$ = 0.048, $\tau_2^2$ = 0.029, $\tau_3^2$ = 0.015; $Q_E$ = 7849.263, $df$ = 509, $p$ < 0.001) | | | | | | |
| **Mechanical induction** | **0.154** | **0.043** | **3.604** | **< 0.001** | **0.070** | **0.237** |
| **Herbivore induction** | **0.150** | **0.042** | **3.577** | **< 0.001** | **0.068** | **0.233** |
| Chemical induction | 0.099 | 0.066 | 1.490 | 0.136 | -0.031 | 0.228 |
| Joint induction | 0.119 | 0.075 | 1.593 | 0.111 | -0.027 | 0.266 |
| Model 12: Effect size (ln*RR*) ~ Induction method × Woodiness | | | | | | |
| ($\tau_1^2$ = 0.053, $\tau_2^2$ = 0.029, $\tau_3^2$ = 0.016; $Q_E$ = 7511.707, $df$ = 505, $p$ < 0.001) | | | | | | |
| **Mechanical induction – Woody** | **0.140** | **0.048** | **2.891** | **0.004** | **0.045** | **0.235** |
| **Herbivore induction – Woody** | **0.168** | **0.048** | **3.472** | **< 0.001** | **0.073** | **0.262** |
| Chemical induction – Woody | 0.109 | 0.080 | 1.360 | 0.174 | -0.048 | 0.267 |
| Joint induction – Woody | 0.116 | 0.125 | 0.929 | 0.353 | -0.129 | 0.361 |
| **Mechanical induction – Non-woody** | **0.207** | **0.099** | **2.090** | **0.037** | **0.013** | **0.400** |
| Herbivore induction – Non-woody | 0.098 | 0.097 | 1.005 | 0.315 | -0.093 | 0.289 |
| Chemical induction – Non-woody | 0.095 | 0.122 | 0.774 | 0.439 | -0.145 | 0.335 |
| Joint induction – Non-woody | 0.122 | 0.111 | 1.102 | 0.270 | -0.095 | 0.340 |
| Model 13: Effect size (ln*RR*) ~ Mechanical induction | | | | | | |

| | | | | | | |
|---|---|---|---|---|---|---|
| ($\tau_1^2 = 0.020$, $\tau_2^2 = 0.014$, $\tau_3^2 = 0.032$; $Q_E = 891.160$, $df = 198$, $p < 0.001$) | | | | | | |
| **Defoliation** | **0.127** | **0.055** | **2.298** | **0.022** | **0.019** | **0.236** |
| Pruning | 0.132 | 0.078 | 1.689 | 0.091 | -0.021 | 0.286 |
| Punching | 0.149 | 0.148 | 1.005 | 0.315 | -0.142 | 0.440 |
| Model 14: Effect size (ln*RR*) ~ Mechanical induction × Woodiness | | | | | | |
| ($\tau_1^2 = 0.007$, $\tau_2^2 = 0.014$, $\tau_3^2 = 0.039$; $Q_E = 792.158$, $df = 196$, $p < 0.001$) | | | | | | |
| **Defoliation – Woody** | **0.174** | **0.061** | **2.875** | **0.004** | **0.055** | **0.293** |
| **Pruning – Woody** | **0.142** | **0.071** | **2.012** | **0.044** | **0.004** | **0.281** |
| Punching – Woody | 0.007 | 0.163 | 0.041 | 0.968 | -0.314 | 0.327 |
| Defoliation – Non-woody | -0.030 | 0.092 | -0.321 | 0.748 | -0.209 | 0.150 |
| Pruning – Non-woody | NA | NA | NA | NA | NA | NA |
| **Punching – Non-woody** | **0.596** | **0.291** | **2.050** | **0.040** | **0.026** | **1.166** |
| Model 15: Effect size (ln*RR*) ~ Herbivore induction | | | | | | |
| ($\tau_1^2 = 0.084$, $\tau_2^2 = 0.038$, $\tau_3^2 = 0.002$; $Q_E = 5574.320$, $df = 232$, $p < 0.001$) | | | | | | |
| **Generalist** | **0.153** | **0.057** | **2.667** | **0.008** | **0.041** | **0.265** |
| **Specialist** | **0.178** | **0.066** | **2.704** | **0.007** | **0.049** | **0.308** |

| | | | | | | |
|---|---|---|---|---|---|---|
| Model 16: Effect size (ln*RR*) ~ Herbivore induction × Woodiness | | | | | | |
| ($\tau_1^2$ = 0.087, $\tau_2^2$ = 0.038, $\tau_3^2$ = 0.003; $Q_E$ = 5532.596, *df* = 230, $p$ < 0.001) | | | | | | |
| **Generalist – Woody** | **0.160** | **0.065** | **2.464** | **0.014** | **0.033** | **0.287** |
| **Specialist – Woody** | **0.155** | **0.072** | **2.144** | **0.032** | **0.013** | **0.297** |
| Generalist – Non-woody | 0.130 | 0.137 | 0.947 | 0.343 | -0.139 | 0.398 |
| Specialist – Non-woody | 0.329 | 0.182 | 1.804 | 0.071 | -0.029 | 0.686 |
| Model 17: Effect size (ln*RR*) ~ Chemical induction | | | | | | |
| ($\tau_1^2$ = 0.002, $\tau_2^2$ = 0.022, $\tau_3^2$ = 0.000; $Q_E$ = 907.119, *df* = 48, $p$ < 0.001) | | | | | | |
| **JA** | **0.105** | **0.035** | **2.997** | **0.003** | **0.036** | **0.174** |
| SA | -0.110 | 0.085 | -1.292 | 0.196 | -0.276 | 0.057 |
| Model 18: Effect size (ln*RR*) ~ Chemical induction × Woodiness | | | | | | |
| ($\tau_1^2$ = 0.005, $\tau_2^2$ = 0.021, $\tau_3^2$ = 0.000; $Q_E$ = 904.526, *df* = 46, $p$ < 0.001) | | | | | | |
| **JA – Woody** | **0.108** | **0.049** | **2.213** | **0.027** | **0.012** | **0.204** |
| SA – Woody | 0.035 | 0.162 | 0.219 | 0.827 | -0.282 | 0.353 |
| JA – Non-woody | 0.105 | 0.070 | 1.499 | 0.134 | -0.032 | 0.243 |
| SA – Non-woody | -0.180 | 0.110 | -1.630 | 0.103 | -0.396 | 0.036 |

| | | | | | | |
|---|---|---|---|---|---|---|
| Model 19: Effect size (ln*RR*) ~ Joint induction | | | | | | |
| ($\tau_1^2 = 0.000$, $\tau_2^2 = 0.005$, $\tau_3^2 = 0.091$; $Q_E = 121.906$, $df = 25$, $p < 0.001$) | | | | | | |
| Defoliation + Herbivore | 0.165 | 0.306 | 0.538 | 0.591 | -0.435 | 0.765 |
| Defoliation + Salivation | -0.004 | 0.221 | -0.019 | 0.985 | -0.437 | 0.429 |
| Defoliation + JA | 0.058 | 0.125 | 0.462 | 0.644 | -0.187 | 0.302 |
| Model 20: Effect size (ln*RR*) ~ Joint induction × Woodiness | | | | | | |
| ($\tau_1^2 = 0.000$, $\tau_2^2 = 0.005$, $\tau_3^2 = 0.111$; $Q_E = 108.547$, $df = 23$, $p < 0.001$) | | | | | | |
| Defoliation + Herbivore – Woody | 0.165 | 0.338 | 0.488 | 0.626 | -0.497 | 0.826 |
| Defoliation + Salivation – Woody | 0.200 | 0.352 | 0.568 | 0.570 | -0.490 | 0.890 |
| Defoliation + JA – Woody | -0.033 | 0.278 | -0.117 | 0.907 | -0.578 | 0.513 |
| Defoliation + Herbivore – Non-woody | NA | NA | NA | NA | NA | NA |
| Defoliation + Salivation – Non-woody | -0.186 | 0.336 | -0.555 | 0.579 | -0.844 | 0.472 |
| Defoliation + JA – Non-woody | 0.088 | 0.156 | 0.562 | 0.574 | -0.218 | 0.394 |

**Table S5 Pairwise contrasts among moderator levels from the multilevel REML meta-analytic models.** Each row reports a Wald contrast between the two levels named in the Test column. The Estimate is the difference in estimated mean effect size (ln*RR*) between the first and the second level (first−second); positive values indicate greater induction in the first level. SE is the standard error of the contrast, z is the Wald statistic, and *p* is the two-sided *p*-value. Contrasts were considered statistically significant when $p < 0.05$, corresponding to 95% confidence intervals (CI) that exclude zero. NA indicates that a contrast was not estimable (e.g., insufficient data for one level).

| Test | Estimate | SE | *z* | *p* |
|---|---|---|---|---|
| Model 2: Effect size (ln*RR*) ~ Woodiness | | | | |
| Woody vs Non-woody | 0.012 | 0.086 | 0.133 | 0.894 |
| Model 3: Effect size (ln*RR*) ~ Leaf tannin group | | | | |
| Total tannins vs Condensed tannins | -0.016 | 0.075 | -0.215 | 0.830 |
| Total tannins vs Hydrolysable tannins | 0.098 | 0.090 | 1.086 | 0.277 |
| Condensed tannins vs Hydrolysable tannins | 0.082 | 0.063 | 1.290 | 0.197 |
| Model 4: Effect size (ln*RR*) ~ Leaf tannin group × Woodiness | | | | |
| Total tannins: Woody vs Non-woody | -0.104 | 0.142 | -0.729 | 0.466 |
| Condensed tannins: Woody vs Non-woody | 0.062 | 0.115 | 0.539 | 0.590 |
| Hydrolysable tannins: Woody vs Non-woody | NA | NA | NA | NA |

| | | | | |
|---|---|---|---|---|
| Model 5: Effect size (ln*RR*) ~ Leaf damage status | | | | |
| Mixed leaves vs Damaged leaves | 0.045 | 0.092 | 0.489 | 0.625 |
| Mixed leaves vs Undamaged leaves | 0.083 | 0.090 | 0.924 | 0.356 |
| Damaged leaves vs Undamaged leaves | 0.038 | 0.042 | 0.910 | 0.363 |
| Model 6: Effect size (ln*RR*) ~ Leaf damage status × Woodiness | | | | |
| Mixed leaves: Woody vs Non-woody | 0.210 | 0.175 | 1.203 | 0.229 |
| Damaged leaves: Woody vs Non-woody | -0.069 | 0.154 | -0.450 | 0.653 |
| Undamaged leaves: Woody vs Non-woody | 0.002 | 0.139 | 0.015 | 0.988 |
| Model 7: Effect size (ln*RR*) ~ Leaf cohort | | | | |
| Mixed leaves vs Preexisting leaves | 0.042 | 0.088 | 0.475 | 0.634 |
| Mixed leaves vs Regrowth leaves | 0.101 | 0.096 | 1.051 | 0.293 |
| Preexisting leaves vs Regrowth leaves | 0.059 | 0.056 | 1.058 | 0.290 |
| Model 8: Effect size (ln*RR*) ~ Leaf cohort × Woodiness | | | | |
| Mixed leaves: Woody vs Non-woody | 0.106 | 0.200 | 0.529 | 0.597 |
| Preexisting leaves: Woody vs Non-woody | -0.036 | 0.098 | -0.365 | 0.715 |
| Regrowth leaves: Woody vs Non-woody | NA | NA | NA | NA |

| | | | | |
|---|---|---|---|---|
| Model 9: Effect size (ln*RR*) ~ Life span | | | | |
| Perennial plants vs Annual plants | 0.141 | 0.120 | 1.171 | 0.241 |
| Model 10: Effect size (ln*RR*) ~ Life span × Woodiness | | | | |
| Perennial plants: Woody vs Non-woody | -0.045 | 0.096 | -0.465 | 0.642 |
| Annual plants: Woody vs Non-woody | NA | NA | NA | NA |
| Model 11: Effect size (ln*RR*) ~ Induction method | | | | |
| Mechanical induction vs Chemical induction | 0.055 | 0.068 | 0.803 | 0.422 |
| Mechanical induction vs Herbivore induction | 0.003 | 0.044 | 0.074 | 0.941 |
| Mechanical induction vs Joint induction | 0.034 | 0.076 | 0.451 | 0.652 |
| Herbivore induction vs Chemical induction | 0.052 | 0.065 | 0.790 | 0.430 |
| Herbivore induction vs Joint induction | 0.031 | 0.074 | 0.417 | 0.677 |
| Joint induction vs Chemical induction | 0.021 | 0.089 | 0.235 | 0.814 |
| Model 12: Effect size (ln*RR*) ~ Induction method × Woodiness | | | | |
| Mechanical induction: Woody vs Non-woody | -0.067 | 0.109 | -0.610 | 0.542 |
| Herbivore induction: Woody vs Non-woody | 0.070 | 0.108 | 0.644 | 0.520 |
| Chemical induction: Woody vs Non-woody | 0.015 | 0.145 | 0.101 | 0.919 |

| | | | | |
|---|---|---|---|---|
| Joint induction: Woody vs Non-woody | -0.006 | 0.165 | -0.038 | 0.970 |
| Model 13: Effect size (ln*RR*) ~ Mechanical induction | | | | |
| Defoliation vs Pruning | -0.005 | 0.096 | -0.053 | 0.958 |
| Defoliation vs Punching | -0.022 | 0.158 | -0.139 | 0.890 |
| Pruning vs Punching | -0.017 | 0.168 | -0.101 | 0.920 |
| Model 14: Effect size (ln*RR*) ~ Mechanical induction × Woodiness | | | | |
| Defoliation: Woody vs Non-woody | 0.204 | 0.110 | 1.859 | 0.063 |
| Pruning: Woody vs Non-woody | NA | NA | NA | NA |
| Punching: Woody vs Non-woody | -0.589 | 0.333 | -1.767 | 0.077 |
| Model 15: Effect size (ln*RR*) ~ Herbivore induction | | | | |
| Generalist vs Specialist | -0.026 | 0.058 | -0.441 | 0.659 |
| Model 16: Effect size (ln*RR*) ~ Herbivore induction × Woodiness | | | | |
| Generalist: Woody vs Non-woody | 0.030 | 0.152 | 0.199 | 0.842 |
| Specialist: Woody vs Non-woody | -0.173 | 0.196 | -0.884 | 0.377 |
| Model 17: Effect size (ln*RR*) ~ Chemical induction | | | | |
| **JA vs SA** | **0.215** | **0.089** | **2.404** | **0.016** |

| | | | | |
|---|---|---|---|---|
| Model 18: Effect size (ln*RR*) ~ Chemical induction × Woodiness | | | | |
| JA: Woody vs Non-woody | 0.003 | 0.085 | 0.034 | 0.973 |
| SA: Woody vs Non-woody | 0.215 | 0.196 | 1.098 | 0.272 |
| Model 19: Effect size (ln*RR*) ~ Joint induction | | | | |
| Defoliation + Herbivore vs Defoliation + Salivation | 0.169 | 0.377 | 0.448 | 0.655 |
| Defoliation + Herbivore vs Defoliation + JA | 0.107 | 0.331 | 0.324 | 0.746 |
| Defoliation + Salivation vs Defoliation + JA | -0.062 | 0.254 | -0.244 | 0.807 |
| Model 20: Effect size (ln*RR*) ~ Joint induction × Woodiness | | | | |
| Defoliation + Herbivore: Woody vs Non-woody | NA | NA | NA | NA |
| Defoliation + Salivation: Woody vs Non-woody | 0.386 | 0.486 | 0.794 | 0.427 |
| Defoliation + JA: Woody vs Non-woody | -0.120 | 0.319 | -0.377 | 0.706 |

42

**Table S6 Simple linear regression models for the relationships between effect size (ln*RR*) and key variables (i.e., damage intensity, damage duration and sampling lag).** $\beta$ = slope estimator; SE = standard error; *df* = degrees of freedom; $R^2$ = goodness of fit of the model. Statistical significance was defined as $p < 0.05$.

| Variables | Effect size (ln*RR*) | | | | | |
|---|---|---|---|---|---|---|
| | $\beta$ | SE | *t* | *p* | $R^2$ | *df* |
| Damage intensity (%) | | | | | | |
| **Total plants** | **0.003** | **0.001** | **6.328** | **< 0.001** | **0.137** | **253** |
| **Woody plants** | **0.004** | **0.000** | **8.444** | **< 0.001** | **0.272** | **191** |
| Non-woody plants | -0.000 | 0.001 | -0.283 | 0.778 | 0.001 | 61 |
| Damage duration (days) | | | | | | |
| Total plants | 0.000 | 0.000 | 0.984 | 0.326 | 0.002 | 406 |
| Woody plants | 0.000 | 0.000 | -0.041 | 0.967 | 0.000 | 300 |
| **Non-woody plants** | **0.006** | **0.002** | **2.972** | **0.004** | **0.078** | **104** |
| Sampling lag (days) | | | | | | |
| Total plants | 0.000 | 0.000 | 1.076 | 0.283 | 0.003 | 429 |
| Woody plants | 0.000 | 0.000 | 0.729 | 0.467 | 0.002 | 319 |

| | | | | | | |
|---|---|---|---|---|---|---|
| Non-woody plants | -0.005 | 0.005 | -1.009 | 0.315 | 0.009 | 108 |

**Table S7 Restricted maximum likelihood (REML) multilevel meta-analytic models for subgroup analyses in woody and non-woody plants.** Models were fitted separately for woody and non-woody subsets. Moderator variables with 95% confidence intervals (CIs) that do not cross zero indicate statistical significance. LCI = 95% lower CI, UCI = 95% upper CI. Effect sizes (ln*RR*) are the response variable of the REML model and the estimate represents the ln*RR* estimated value for the corresponding variable in the model. Variance components: $\tau_1^2$ = between-study variance (random intercept for publication ID), $\tau_2^2$ = within-study variance among effect sizes (random intercept for effect ID nested within publication), and $\tau_3^2$ = between-species variance (random intercept for species ID, cross-classified across studies). $Q_E$ = test statistic for residual heterogeneity (Cochran's Q) for the fitted model. **Note:** For some subgroup models, a moderator may be non-estimable when it has only one level within a subset (e.g., life span in woody plants if all woody observations are perennial).

| Moderator variables | Estimate | *SE* | *z* | *p* | LCI | UCI |
|---|---|---|---|---|---|---|
| Model 21: Effect size (ln*RR*) ~ Leaf tannin group (Woody) | | | | | | |
| ($\tau_1^2 = 0.056$, $\tau_2^2 = 0.033$, $\tau_3^2 = 0.000$; $Q_E = 3852.552$, $df = 364$, $p < 0.001$) | | | | | | |
| **Total tannins** | **0.158** | **0.065** | **2.452** | **0.014** | **0.032** | **0.285** |
| **Condensed tannins** | **0.145** | **0.042** | **3.419** | **< 0.001** | **0.062** | **0.229** |
| **Hydrolysable tannins** | **0.220** | **0.067** | **3.291** | **< 0.001** | **0.089** | **0.352** |
| Model 22: Effect size (ln*RR*) ~ Leaf tannin group (Non-woody) | | | | | | |
| ($\tau_1^2 = 0.043$, $\tau_2^2 = 0.017$, $\tau_3^2 = 0.057$; $Q_E = 2727.350$, $df = 144$, $p < 0.001$) | | | | | | |

| | | | | | | |
|---|---|---|---|---|---|---|
| Total tannins | 0.161 | 0.133 | 1.213 | 0.225 | -0.099 | 0.421 |
| Condensed tannins | 0.082 | 0.132 | 0.623 | 0.533 | -0.177 | 0.342 |
| Model 23: Effect size (ln*RR*) ~ Leaf damage status (Woody) | | | | | | |
| ($\tau_1^2 = 0.055$, $\tau_2^2 = 0.033$, $\tau_3^2 = 0.000$; $Q_E = 3640.266$; $df = 364$; $p < 0.001$) | | | | | | |
| **Mixed leaves** | **0.315** | **0.100** | **3.160** | **0.002** | **0.120** | **0.511** |
| **Damaged leaves** | **0.160** | **0.044** | **3.638** | **< 0.001** | **0.074** | **0.246** |
| **Undamaged leaves** | **0.125** | **0.041** | **3.041** | **0.002** | **0.044** | **0.205** |
| Model 24: Effect size (ln*RR*) ~ Leaf damage status (Non-woody) | | | | | | |
| ($\tau_1^2 = 0.041$, $\tau_2^2 = 0.017$, $\tau_3^2 = 0.061$; $Q_E = 2704.703$; $df = 143$; $p < 0.001$) | | | | | | |
| Mixed leaves | 0.068 | 0.162 | 0.422 | 0.673 | -0.249 | 0.386 |
| Damaged leaves | 0.285 | 0.198 | 1.443 | 0.149 | -0.102 | 0.673 |
| Undamaged leaves | 0.073 | 0.143 | 0.507 | 0.612 | -0.208 | 0.353 |
| Model 25: Effect size (ln*RR*) ~ Leaf cohort (Woody) | | | | | | |
| ($\tau_1^2 = 0.050$, $\tau_2^2 = 0.033$, $\tau_3^2 = 0.000$; $Q_E = 3688.960$; $df = 364$; $p < 0.001$) | | | | | | |
| Mixed leaves | 0.194 | 0.085 | 2.292 | 0.022 | 0.028 | 0.360 |
| **Preexisting leaves** | **0.134** | **0.040** | **3.346** | **< 0.001** | **0.056** | **0.213** |

| | | | | | | |
|---|---|---|---|---|---|---|
| **Regrowth leaves** | **0.201** | **0.054** | **3.716** | **< 0.001** | **0.095** | **0.307** |
| Model 26: Effect size (ln*RR*) ~ Leaf cohort (Non-woody) | | | | | | |
| ($\tau_1^2$ = 0.043, $\tau_2^2$ = 0.017, $\tau_3^2$ = 0.056; $Q_E$ = 4051.150; *df* = 144; $p < 0.001$) | | | | | | |
| Mixed leaves | 0.011 | 0.182 | 0.059 | 0.953 | -0.346 | 0.368 |
| Preexisting leaves | 0.159 | 0.107 | 1.486 | 0.137 | -0.051 | 0.369 |
| Model 27: Effect size (ln*RR*) ~ Life span (Woody) | | | | | | |
| NA | NA | NA | NA | NA | NA | NA |
| Model 28: Effect size (ln*RR*) ~ Life span (Non-woody) | | | | | | |
| ($\tau_1^2$ = 0.044, $\tau_2^2$ = 0.017, $\tau_3^2$ = 0.057; $Q_E$ = 2794.592; *df* = 144; $p < 0.001$) | | | | | | |
| Annual | 0.069 | 0.158 | 0.440 | 0.660 | -0.240 | 0.379 |
| Perennial | 0.144 | 0.108 | 1.334 | 0.182 | -0.068 | 0.357 |
| Model 29: Effect size (ln*RR*) ~ Induction method (Woody) | | | | | | |
| ($\tau_1^2$ = 0.055, $\tau_2^2$ = 0.033, $\tau_3^2$ = 0.000; $Q_E$ = 3854.490; *df* = 363; $p < 0.001$) | | | | | | |
| **Mechanical** | **0.146** | **0.045** | **3.214** | **0.001** | **0.057** | **0.235** |
| Chemical | 0.115 | 0.073 | 1.568 | 0.117 | -0.029 | 0.259 |
| **Herbivore** | **0.184** | **0.043** | **4.291** | **< 0.001** | **0.100** | **0.268** |

| | | | | | | |
|---|---|---|---|---|---|---|
| Joint | 0.129 | 0.122 | 1.062 | 0.288 | -0.109 | 0.368 |
| Model 30: Effect size (ln*RR*) ~ Induction method (Non-woody) | | | | | | |
| ($\tau_1^2$ = 0.050, $\tau_2^2$ = 0.017, $\tau_3^2$ = 0.059; $Q_E$ = 3657.216; *df* = 142; *p* < 0.001) | | | | | | |
| Mechanical | 0.211 | 0.109 | 1.934 | 0.053 | -0.003 | 0.424 |
| Chemical | 0.084 | 0.134 | 0.629 | 0.529 | -0.179 | 0.347 |
| Herbivore | 0.064 | 0.109 | 0.583 | 0.560 | -0.150 | 0.277 |
| Joint | 0.117 | 0.115 | 1.019 | 0.308 | -0.108 | 0.343 |
| Model 31: Effect size (ln*RR*) ~ Mechanical induction (Woody) | | | | | | |
| ($\tau_1^2$ = 0.017, $\tau_2^2$ = 0.015, $\tau_3^2$ = 0.032; $Q_E$ = 711.925; *df* = 149; *p* < 0.001) | | | | | | |
| **Defoliation** | **0.177** | **0.063** | **2.821** | **0.005** | **0.054** | **0.299** |
| Pruning | 0.134 | 0.077 | 1.746 | 0.081 | -0.016 | 0.284 |
| Punching | 0.006 | 0.168 | 0.035 | 0.972 | -0.323 | 0.335 |
| Model 32: Effect size (ln*RR*) ~ Mechanical induction (Non-woody) | | | | | | |
| ($\tau_1^2$ = 0.000, $\tau_2^2$ = 0.003, $\tau_3^2$ = 0.030; $Q_E$ = 80.233; *df* = 47; *p* = 0.002) | | | | | | |
| Defoliation | -0.048 | 0.065 | -0.734 | 0.463 | -0.175 | 0.079 |
| **Punching** | **0.596** | **0.240** | **2.487** | **0.013** | **0.126** | **1.066** |

| | | | | | | |
|---|---|---|---|---|---|---|
| Model 33: Effect size (ln*RR*) ~ Herbivore induction (Woody) | | | | | | |
| ($\tau_1^2$ = 0.076, $\tau_2^2$ = 0.049, $\tau_3^2$ = 0.002; $Q_E$ = 2179.408; *df* = 170; $p < 0.001$) | | | | | | |
| **Generalist** | **0.156** | **0.063** | **2.470** | **0.014** | **0.032** | **0.279** |
| **Specialist** | **0.156** | **0.072** | **2.160** | **0.031** | **0.014** | **0.297** |
| Model 34: Effect size (ln*RR*) ~ Herbivore induction (Non-woody) | | | | | | |
| ($\tau_1^2$ = 0.055, $\tau_2^2$ = 0.018, $\tau_3^2$ = 0.055; $Q_E$ = 3353.188; *df* = 60; $p < 0.001$) | | | | | | |
| Generalist | 0.127 | 0.146 | 0.874 | 0.382 | -0.158 | 0.413 |
| Specialist | 0.342 | 0.176 | 1.939 | 0.052 | -0.004 | 0.688 |
| Model 35: Effect size (ln*RR*) ~ Chemical induction (Woody) | | | | | | |
| ($\tau_1^2$ = 0.000, $\tau_2^2$ = 0.026, $\tau_3^2$ = 0.010; $Q_E$ = 889.506; *df* = 27; $p < 0.001$) | | | | | | |
| **JA** | **0.130** | **0.059** | **2.178** | **0.029** | **0.013** | **0.246** |
| SA | 0.048 | 0.175 | 0.272 | 0.785 | -0.295 | 0.390 |
| Model 36: Effect size (ln*RR*) ~ Chemical induction (Non-woody) | | | | | | |
| ($\tau_1^2$ = 0.000, $\tau_2^2$ = 0.000, $\tau_3^2$ = 0.000; $Q_E$ = 15.020; *df* = 19; $p = 0.721$) | | | | | | |
| **JA** | **0.110** | **0.044** | **2.474** | **0.013** | **0.023** | **0.197** |
| **SA** | **-0.154** | **0.064** | **-2.405** | **0.016** | **-0.280** | **-0.029** |

| | | | | | | |
|---|---|---|---|---|---|---|
| Model 37: Effect size (ln*RR*) ~ Joint induction (Woody) | | | | | | |
| ($\tau_1^2$ = 0.000, $\tau_2^2$ = 0.000, $\tau_3^2$ = 0.000; $Q_E$ = 8.764; *df* = 11; *p* = 0.644) | | | | | | |
| **Defoliation + Herbivore** | **0.145** | **0.009** | **16.293** | **< 0.001** | **0.127** | **0.162** |
| Defoliation + JA | -0.090 | 0.118 | -0.770 | 0.441 | -0.321 | 0.140 |
| Defoliation + Salivation | 0.198 | 0.107 | 1.838 | 0.066 | -0.013 | 0.408 |
| Model 38: Effect size (ln*RR*) ~ Joint induction (Non-woody) | | | | | | |
| ($\tau_1^2$ = 0.000, $\tau_2^2$ = 0.006, $\tau_3^2$ = 0.129; $Q_E$ = 99.783; *df* = 12; *p* < 0.001) | | | | | | |
| Defoliation + JA | 0.089 | 0.167 | 0.533 | 0.594 | -0.239 | 0.417 |
| Defoliation + Salivation | -0.187 | 0.362 | -0.517 | 0.605 | -0.895 | 0.522 |

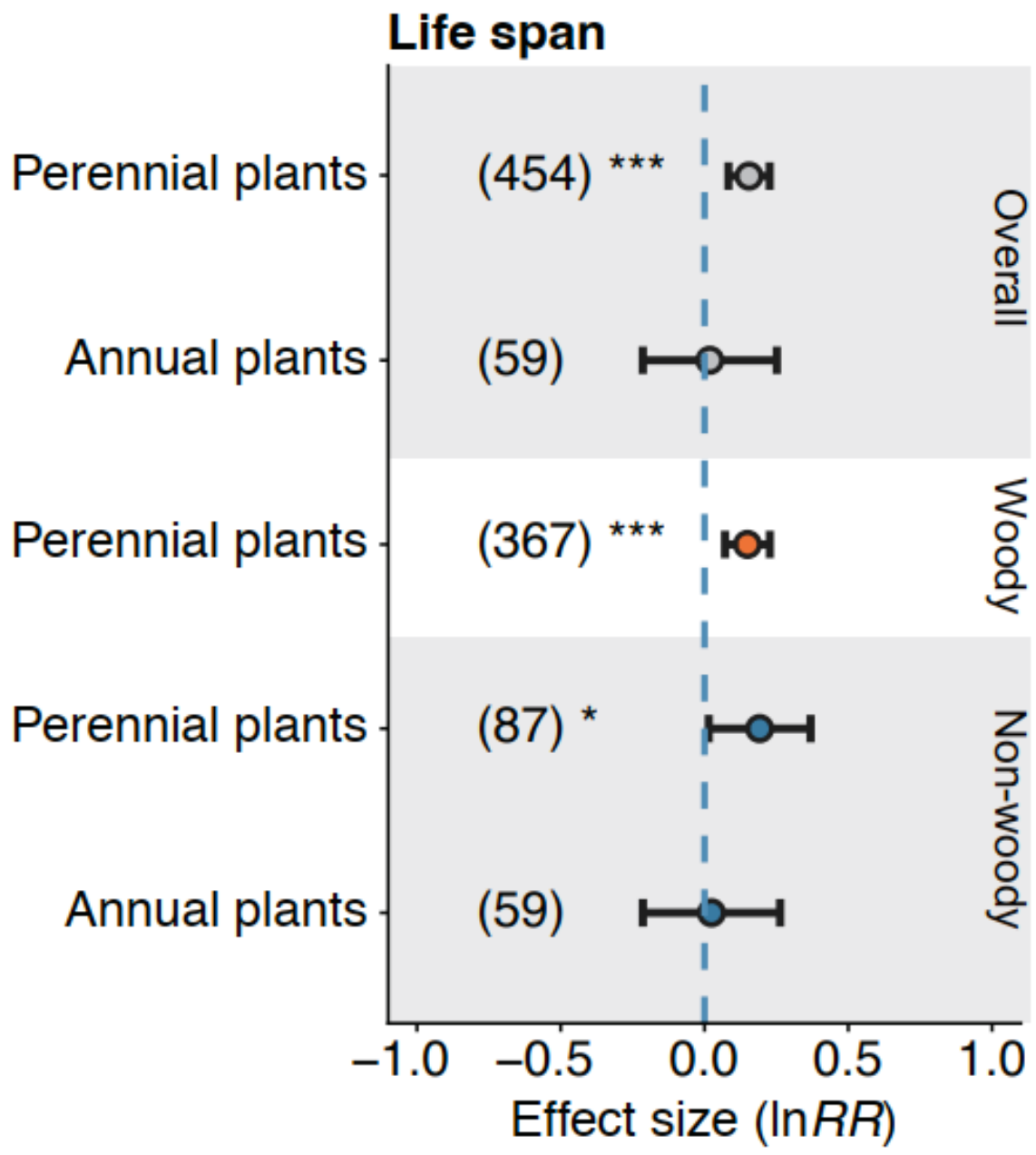


**Figure S1 Life span as a categorical moderator of induced leaf tannin responses.** The plot shows multilevel REML estimates of effect size (ln*RR*) with 95% confidence intervals (horizontal lines) for annual versus perennial plants, reported for the full dataset (Overall) and stratified by woodiness (woody vs. non-woody). The dashed vertical line marks ln*RR* = 0; confidence intervals entirely above zero indicate significantly positive induction, whereas intervals overlapping zero indicate no detectable effect. Numbers in parentheses denote the number of effect sizes contributing to each estimate, and asterisks indicate statistical significance (*$p < 0.05$, ***$p < 0.001$).

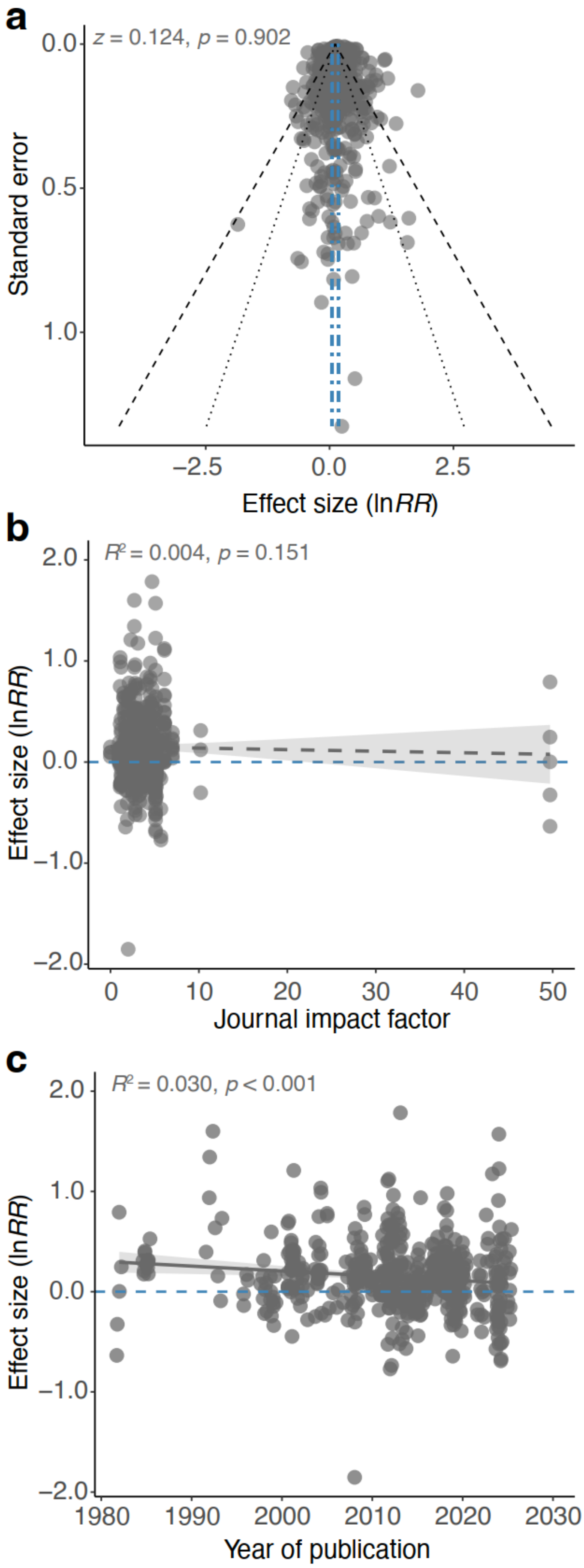
a
z = 0.124, p = 0.902
Standard error
0.0
0.5
1.0
−2.5
0.0
2.5
Effect size (lnRR)
b
R² = 0.004, p = 0.151
2.0
1.0
0.0
−1.0
−2.0
Effect size (lnRR)
0
10
20
30
40
50
Journal impact factor
c
R² = 0.030, p < 0.001
2.0
1.0
0.0
−1.0
−2.0
Effect size (lnRR)
1980
1990
2000
2010
2020
2030
Year of publication

**Figure S2 Tests for publication bias in the dataset. (a)** Funnel plot of ln*RR* against standard error, with guidelines showing expected limits around the grand mean. The p-value refers to Egger's regression test; $p > 0.05$ indicates no evidence of funnel plot asymmetry. **(b–c)** Relationships of ln*RR* with journal impact factor **(b)** and publication year **(c)**; *p*-values refer to the corresponding regressions, and non-significant fits are shown with dashed lines.
